# Pre-reproductive stress in adolescent female rats alters oocyte microRNA expression and offspring phenotypes: pharmacological interventions and putative mechanisms

**DOI:** 10.1038/s41398-021-01220-1

**Published:** 2021-02-05

**Authors:** Hiba Zaidan, Dalia Galiani, Inna Gaisler-Salomon

**Affiliations:** 1 School of Psychological Sciences and the Integrated Brain and Behavior Research Center, University of Haifa, Haifa, Israel; 2 Department of Biological Regulation, Weizmann Institute of Science, Rehovot, Israel

**Keywords:** Molecular neuroscience, Epigenetics and behaviour

## Abstract

Pre-reproductive stress (PRS) to adolescent female rats alters anxiogenic behavior in first (F1)- and second-generation (F2) offspring and increases mRNA expression of corticotropin-releasing factor receptor type 1 (*Crhr1)* in oocytes and in neonate offspring brain. Here, we ask whether the expression of *Crhr1* and *Crhr1-*targeting microRNA is altered in brain, blood, and oocytes of exposed females and in the brain of their neonate and adult F1 and F2 offspring. In addition, we inquire whether maternal post-stress drug treatment reverses PRS-induced abnormalities in offspring. We find that PRS induces a selective increase in *Crhr1-*targeting mir-34a and mir-34c in blood and oocytes, while non-*Crhr1* microRNA molecules remain unaltered. PRS induces similar microRNA changes in prefrontal cortex of F1 and F2 neonates. In adult animals, cortical *Crhr1*, but not mir-34, expression is affected by both maternal and direct stress exposure. Post-PRS fluoxetine (FLX) treatment increases pup mortality, and both FLX and the *Crhr1* antagonist NBI 27914 reverse some of the effects of PRS and also have independent effects on F1 behavior and gene expression. PRS also alters behavior as well as gene and miRNA expression patterns in paternally derived F2 offspring, producing effects that are different from those previously found in maternally derived F2 offspring. These findings extend current knowledge on inter- and trans-generational transfer of stress effects, point to microRNA changes in stress-exposed oocytes as a potential mechanism, and highlight the consequences of post-stress pharmacological interventions in adolescence.

## Introduction

Exposure to an unpredictable, adverse environment has long-term effects on health, behavior, and endocrine function. Human and animal studies show that the effects of stress during or prior to gestation can propagate onto future generations^1–4^, and impact behavior and hypothalamic–pituitary–adrenal (HPA) axis function in offspring^5^. The influences of early life stress on offspring behavior and neuroendocrine function have been reported for up to four generations^6–11^. The trans-generational effects of stress during adolescence, a period of profound changes to brain structure and function^12,13^, have been less extensively studied.

Different mechanisms have been proposed to account for the transfer of information across generations, e.g., effects of environmental factors on the uterine environment, maternal care, and the epigenome^14–16^. Notably, while uterine changes and maternal care are likely to impact first-generation (F1) offspring, they are unable to account for changes observed in second- and third-generation offspring (F2 and F3, respectively), or for effects transmitted via the paternal lineage^16–19^. Epigenetic alterations in sperm were shown in several studies and may provide a biological mechanism for transmission^20–25^. In particular, changes in microRNA (miRNA) expression were proposed to occur after stress exposure in the parental generation and to account for behavioral changes in offspring^26,20^. Notably, the majority of studies on trans-generational stress effects have been performed in male rodents. Epigenetic changes in oocytes as mediators of inter- and trans-generational transmission remain poorly understood^27^. In particular, microRNA changes in oocytes have not been investigated as agents of inter- or trans-generational transmission of stress effects to date.

In a recent series of studies, we investigated the impact of mild, chronic pre-reproductive stress (PRS) to adolescent female rats on F1 and F2 offspring behavior, HPA axis function, prefrontal cortex (PFC) morphology, and gene expression patterns^28–31^. We observed changes in behavioral assays measuring fear and anxiety in F1 and maternally derived F2 offspring, changes in corticosterone (CORT) levels in exposed females and offspring, and alterations in expression of corticotropin-releasing factor receptor type 1 (*Crhr1*), which plays a central role in the HPA axis response to stress^28,29^, in the PFC of exposed females and in the brain of their neonate offspring. Interestingly, *Crhr1* levels in PRS-exposed female oocytes were substantially elevated, and were also increased in adult offspring PFC. Moreover, cortical *Crhr1* expression depended on maternal as well as offspring exposure to stress.

Here, we first seek to determine whether PRS to adolescent female rats affects the expression of *Crhr1-*targeting miRNAs in brain, blood, and oocytes. Relying on a miRNA database search and previous studies, and are some of a few miRNA molecules, which have been implicated on the stress response^32–36^, target *Crhr1*^32,37–40^, and are some of a few miRNA molecules expressed in oocytes^41^. Second, we ask whether maternal PRS affects *Crhr1* and mir-34 expression in neonate F1 and F2 offspring, and alters expression patterns in animals that have been exposed to low- and high-stress conditions. Third, we inquire whether post-PRS, pre-gestational pharmacological treatment can reverse the impact of PRS on offspring behavioral and molecular phenotypes. Specifically, we test the impact of the CRHR1 antagonist NBI-27914 (NBI)^42^ and the antidepressant fluoxetine (FLX), a selective serotonin reuptake inhibitor (SSRI) commonly prescribed to treat stress-related psychopathology in adolescence^43,44^ and previously shown to reverse the effects of chronic unpredictable stress in animal models^45,46^. Finally, since paternally derived offspring are less likely to be affected by the uterine environment and maternal care, we study PRS-induced changes in behavior and mRNA/miRNA expression in F2 rats derived from F1 males, and compare them to our previous findings in maternally derived F2 offspring^28,29^.

## Methods

Detailed methods and materials are provided in the Supplementary information (SI).

### Animals

Adolescent female Sprague-Dawley rats and the adult males used for mating were purchased from Envigo (Jerusalem). The study was approved by the University of Haifa Committee on animal experimentation (294/13, 351/14, 711/20). Animal care and experiments were performed in line with NIH guidelines and regulations.

### Experimental procedure

**Fig. 1:**
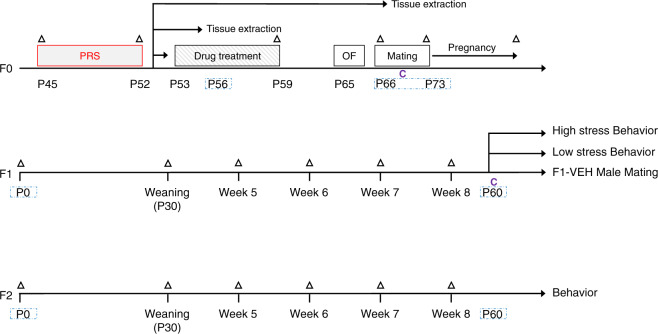
Experimental procedure. Timeline of the experimental procedure in the parent (F0) and offspring (F1 and F2) generations. Triangle: determination of weight. Blue dashed rectangle: assessment of gene expression and/or miRNA levels. C: assessment of CORT levels.

The experimental timeline is outlined in Fig. 1 and described in detail in the Supplementary information (SI). Briefly, adolescent (P45) female rats (F0) were randomly assigned to control (C) or PRS groups. PRS rats were exposed to a 7-day chronic unpredictable stress (CUS) protocol^28–31,47^. The 7-day procedure included (1) 15 min warm swim (22 °C), (2) 10 min cold swim (15 °C) followed by warming (3) 24 h food and water deprivation, (4) 24 h constant light, (5) 3 times 30 min on a raised platform at 30 min intervals, (6) electric shock (10 × 0.5 mA 1 s at 30 s intervals), (7) 24 h crowding (8 females in a cage 56 × 35 × 19 cm high) with constant light. Rats were then divided into 3 cohorts: P56 (Cohort 1), P66-73 (Cohort 2), and Drug Treatment (Cohort 3). Tissue samples were extracted from rats in Cohorts 1 and 2 on PND 56 and PND 66–73 (the mating period), respectively (see Fig. 1). C and PRS Cohort 3 rats received 5–7 days of intraperitoneal (i.p.) vehicle (VEH), NBI or FLX starting on P53. In F1, we examined male and female offspring of all six groups (F1-C/VEH, F1-C/NBI, F1-C/FLX, F1-PRS/VEH, F1-PRS/NBI, F1-PRS/FLX). To produce the F2 generation, we bred F1-C and F1-PRS behaviorally naïve males with naïve females. In F2, we examined their male and female offspring (F2-C, F2-PRS). Experimental *n*’s are depicted in figures.

### Behavior

F0: Dams were tested for general locomotor abnormalities and novelty-induced anxiogenic behavior in the open field (OF). F1: Male and female adult progeny were randomly divided into low- and high-stress exposure. ‘Low-stress’ rats were tested in the OF followed 24 h later by Novel Object Recognition (NOR). ‘High-stress’ rats were tested in the elevated plus maze (EPM) followed 24 h later by the fear conditioning and extinction test. F2: Male and female adult progeny were tested in the OF, NOR, and social preference (SP) tests. Two weeks later, rats were tested in the EPM followed 24 h later by the fear conditioning and extinction test. Therefore, F2 progeny were all exposed to high-stress behavioral testing. Exclusion Criteria are detailed in Tables S1 and S2. Experimenters were blind to the group allocation during behavioral test performance.

### CORT quantification

Blood was collected in F0 dams post-weaning, and in F1 adult behaviorally naïve males and females. Blood collection was performed in the morning, and CORT was quantified by ELISA as previously described^28^.

### mRNA and miRNA expression analysis

We assessed mRNA and miRNA expression in oocytes and blood of F0 females, and in the AMY and PFC of F0 females and their neonate and adult F1 and F2 offspring. Dissections, RNA and miRNA extraction, cDNA preparation, and quantitative real-time PCR (qRT-PCR) were performed as described previously^29,48,49^ (see Table S3 for primer list). Fold-change values were calculated using the ddCt method^50^ relative to the housekeeping gene hypoxanthine phosphoribosyl transferase (HPRT; mRNA; brain), 18s (mRNA; oocytes), or U6-snRNA/RNU6 (miRNA), which were found to be expressed in similar levels across groups and tissue types.

### Statistical analyses

The sample sizes of each experiment were determined based on our previous studies^28,29^. No randomization was performed. Data were analyzed with Student’s two-sided *t*-tests, analysis of variance (ANOVA), repeated-measures ANOVA, multivariate analysis of variance (MANOVA), Pearson correlation coefficients, and the Chi-Square test of independence (see figure legends and SI for details) using SPSS 23 Statistics software (IBM, Chicago, IL). The least significant difference (LSD) test was used for post-hoc comparisons when interactions were significant. Homogeneity of variance was confirmed with Levene’s test for equality of variances.

## Results

### F0 dams and F1 neonates: basic attributes

Stress to adolescent female rats led to short-term weight loss and long-term weight gain (Fig. S1a), and decreased locomotor activity during the first minute in the OF; drug treatment had no effect on either measure (Fig. S1b). Examining F1 litter attributes, we found no PRS- or drug-induced differences in litter size or in male/female pup ratio. However, maternal FLX or NBI decreased pup weight, and FLX treatment tended to increase pup mortality odds. Pup mortality was particularly high in the F1-PRS/FLX group (Table S4).

### F0 dams, F1 and F2 neonates: mRNA and miRNA expression changes

**Fig. 2:**
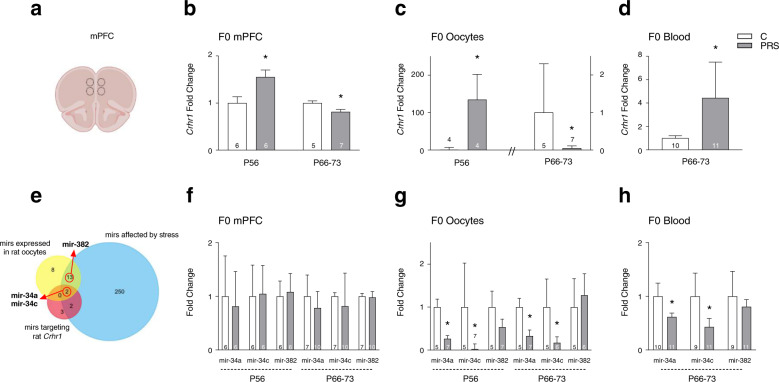
PRS-induced *Crhr1* mRNA and miRNA expression changes in F0 females. In mPFC (**a**), *Crhr1* mRNA is elevated in PRS-exposed rats on P56 (one-way ANOVA, F_1,10_ = 6.2, *p* < 0.05), and decreased to below control levels by P66-73 (**b;** one-way ANOVA, F_1,10_ = 6.9, *p* < 0.05), and a similar pattern is observed in oocytes (**c**; P56: one-way ANOVA, F_1,6_ = 7.0, *p* < 0.05; P66-73: one-way ANOVA, F_1,10_ = 5.91, *p* < 0.01). **d** In blood, *Crhr1* mRNA is increased in PRS-exposed rats at P66-73 (one-way ANOVA, F_1,19_ = 9.138, *p* < 0.01). **e** Venn diagram demonstrating selection of miRNA molecules assessed in current study: mir-34a and mir-34c, but not mir-382, target *Crhr1*. All three miRNAs are involved in the stress response and are expressed in oocytes. **f** No PRS-induced changes in mir-34a, mir-34c, and mir-382 expression in mPFC at either time point. **g** Mir-34a and mir-34c expression is decreased in oocytes of PRS-exposed rats on P56 (one-way ANOVA, F_1,10_ = 10.2, *p* < 0.01, F_1,10_ = 7.2, *p* < 0.05, respectively) and at P66-73 (one-way ANOVA, F_1,10_ = 5.69, *p* < 0.05, F_1,9_ = 5.15, *p* < 0.05, respectively), with no change in mir-382 at either time point. **h** Mir-34a and mir-34c, but not mir-382, expression is decreased in blood of PRS-exposed rats (one-way ANOVA, F_1,19_ = 6.621, *p* < 0.05, F_1,18_ = 4.67, *p* < 0.05, respectively). Data presented as means and standard errors of fold-change, relative to C. ^*^post-hoc/one-way ANOVA *p*’s < 0.05.

Raw dCt values for RT-PCR experiments are presented in Table S5. In F0 dams, we assessed mRNA and miRNA expression on P56 (4 days after stress) and during P66–73 (equivalent to the time of mating, see Fig. 1). In replication of our previous results^29^, we found an increase in *Crhr1* mRNA in mPFC (Fig. 2a) of PRS-exposed female rats 4 days after exposure (P56). This increase was transient; by P66–73, *Crhr1* expression was lower than control levels (Fig. 2b). In oocytes, *Crhr1* mRNA was transiently elevated on P56, decreasing below control levels by P66–73 (Fig. 2c). In blood, *Crhr1* mRNA was elevated at P66–73 (Fig. 2d). PRS had no effect on *Crhr1* expression in the AMY at either time point (Fig. S2a). Expression of CRH receptor type 2 (*Crhr2*) mRNA was not altered by PRS exposure in mPFC or AMY, and was undetected in oocytes or blood (not shown).

Relying on a miRNA database search, we focused our investigation on mir-34a, mir-34c, and mir-382. All three miRNA molecules are expressed in oocytes, the blood stream, and brain^41^ (confirmed by pilot studies in our lab), and participate in the stress response^32–36^; only mir-34a and mir-34c, however, target *Crhr1*^32,37–40^ (Fig. 2e). In mPFC, we found no PRS-induced changes in mir-34a and mir-34c at P56 or P66-73 and no changes in mir-382 expression at either time point (Fig. 2f). In the AMY, PRS had no effect on mir-34a or mir-382, but decreased mir-34c expression at P56 (Fig. S2b). No change in mir-34a, mir-34c, and mir-382 was found in AMY at P66–73 (Fig. S2c). In oocytes, we found a 74% PRS-induced decrease in mir-34a on P56 and a 67% decrease at P66–73, with no change in mir-382 at either time point. Mir-34c decreased by 98% on P56 and 83% at P66–73 (Fig. 2g). A decrease in mir-34a and mir-34c, but no change in mir-382, was also observed in blood (Fig. 2h). Oocytic expression of additional non-*Crhr1*-targeting miRNAs (mir-137-3p, mir-137-5p, mir-203-3p, mir-203-5p, mir-493-3p, and mir-493-5p) was not altered by PRS (Fig. S3).

**Fig. 3:**
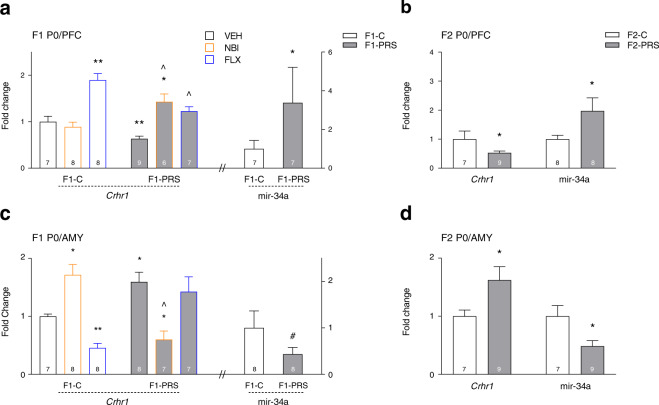
PRS- and maternal drug treatment-induced gene/miRNA expression changes in neonate PFC and AMY of F1 and F2 offspring. **a** Maternal PRS decreases *Crhr1* in PFC of neonate F1 offspring. This effect is normalized by post-PRS maternal treatment with FLX and NBI, which increases *Crhr1* mRNA beyond control levels. Maternal FLX increases *Crhr1* expression in F1-C (2 × 3 ANOVA, group × drug F_2,39_ = 15.1, *p* < 0.00001). Maternal PRS also increases mir-34a expression in F1-VEH PFC (one-way ANOVA, F_1,12_ = 5.6, *p* < 0.05). **b** As in F1-VEH animals, PRS decreases *Crhr1* mRNA (one-way ANOVA, F_1,14_ = 8.1, *p* < 0.05) and increases mir-34a expression (F_1,14_ = 7.7, *p* < 0.05) in PFC of neonate F2 offspring. **c** Maternal PRS increases *Crhr1* mRNA in AMY of neonate F1 offspring. This effect is normalized by post-PRS treatment with NBI, which decreases *Crhr1* mRNA beyond control levels (2 × 3 ANOVA, group × drug F_2,39_ = 29.2, *p* < 0.00001). Maternal PRS also marginally decreases mir-34a expression in F1-VEH AMY (one-way ANOVA, F_1,14_ = 4.002, *p* = 0.065). **d** PRS increases *Crhr1* mRNA (one-way ANOVA, F_1,14_ = 8.04, *p* < 0.05) and decreases mir-34a expression (one-way ANOVA, F_1,14_ = 6.5, *p* < 0.05) in AMY of neonate F2 offspring. Data presented as means and standard errors of fold-change relative to F1-C/VEH (**a**, **c**) or F2-C (**b**, **d**). Post-hoc/one-way ANOVA ^#^*p* < 0.075, *p < 0.05, **p < 0.001, ^*p* < 0.05, post-hoc relative to F1-PRS/VEH.

We next analyzed PRS- and drug-induced changes in *Crhr1* and mir-34a, mir-34c and mir-382 expression in PFC and AMY of F1 and F2 neonates. In F1 PFC (Fig. 3a), maternal PRS decreased *Crhr1* expression, and post-PRS treatment with the CRHR1 antagonist NBI or the SSRI FLX reversed this effect. Maternal FLX also increased *Crhr1* in neonate offspring of Control dams. Mir-34a expression was increased in PFC of F1-PRS neonate offspring. A decrease in *Crhr1* and an increase in mir-34a was similarly observed in neonate F2 offspring (Fig. 3b). In AMY, maternal PRS led to the opposite pattern of *Crhr1* and mir-34a expression: it increased *Crhr1* and decreased mir-34a expression in F1/VEH (Fig. 3c) and F2 neonates (Fig. 3d). In F1 AMY, post-PRS NBI treatment reversed PRS effects, and treatment of Control dams with NBI increased, while FLX treatment decreased, *Crhr1* mRNA. No change was found in mir-34c or mir-382 expression in F1 and F2 PFC or AMY (not shown).

### F1: weight gain in adolescence

As can be seen in Table S6, both male and female F1-PRS/FLX offspring gained less weight than rats in all other conditions.

### F1 adults: behavior changes under low- and high-stress conditions

**Fig. 4:**
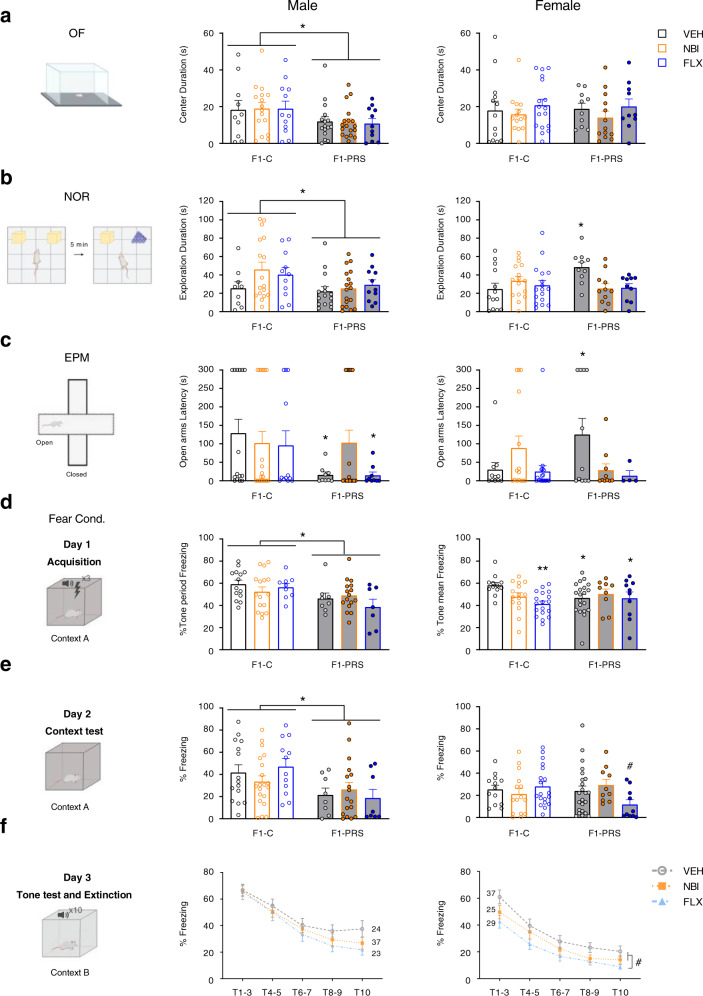
Maternal PRS- and drug-induced changes in F1 offspring behavior under low- and high-stress testing conditions. **a** In the OF (left), male F1-PRS rats spend less time in the center of the OF compared to F1-C, regardless of maternal drug treatment (center**;** 2 × 3 ANOVA, group F_1,83_ = 7.6, *p* < 0.01), while female offspring are unaffected by maternal PRS or drug treatment (right). **b** In the NOR task (left), PRS decreases overall exploration time in males (center; 2 × 3 ANOVA, group, F_1,75_ = 4.45, *p* < 0.05), but increases it in females, and the latter effect is reversed by maternal treatment with either NBI or FLX (right; 2 × 3 ANOVA, group × drug F_2,71_ = 4.7, *p* < 0.05)*.* **c** In the EPM (left), maternal PRS decreases open arm latency in males (2 × 3 ANOVA, group F_1,76_ = 4.9, *p* < 0.05), and subsequent NBI treatment reverses this effect (center; one-way ANOVA on F1-C/VEH and F1-PRS groups, F_3,47_ = 2.9, *p* < 0.05). In female offspring, maternal PRS increases latency, and subsequent treatment with either NBI or FLX reverses this effect (right; one-way ANOVA on F1-C/VEH and F1-PRS groups, F_3,32_ = 2.9, *p* < 0.05). **d** On the Acquisition Day (Day 1) of the fear conditioning/extinction task, (left), maternal PRS decreases freezing during the tone period in male offspring (2 × 3 ANOVA, group F_1,64_ = 9.3, *p* < 0.01), and subsequent drug treatment had no effect (center). In female offspring (right), we observe an interaction that approaches significance between group and drug treatment (2 × 3 ANOVA, F_2,78_ = 3.1, *p* = 0.052), so that maternal PRS and/or FLX decreased freezing during tone presentation. **e** During the Context Test (Day 2) (left), maternal PRS decreases freezing in male offspring, and subsequent drug treatment has no effect (center; 2 × 3 ANOVA, group F_1,74_ = 10.4, *p* < 0.01). In female offspring (right), only PRS followed by FLX marginally decreases freezing (2 × 3 ANOVA, group × drug F_2,84_ = 3.0, *p* = 0.055). **f** During the Tone Test/Extinction (Day 3), gradual extinction of fear is observed in male offspring (repeated-measures ANOVA, tone F_4.4,343.2_ = 25.7, *p* < 0.001), but maternal PRS or drug treatment have no effect on freezing during tones 1–3 (Tone Test) or the subsequent 7 tones (Extinction; center). In female offspring (right), freezing is extinguished over time (repeated-measures ANOVA, tone F_4.3, 370_ = 20.3, *p* < 0.0001), maternal FLX treatment decreases freezing during tones 1–3 (repeated-measures ANOVA, drug F_2,85_ = 4.1, *p* < 0.05), and marginally decreases freezing in tones 4–10 (repeated-measures ANOVA, drug F_2,85_ = 3.0, *p* = 0.055) regardless of maternal PRS exposure. Data presented as individual values, with bars and whiskers representing means and standard errors, respectively (**a–e**), or as means and standard errors (**f**). #*p* < 0.075, *p < 0.05, **p < 0.001, post-hoc relative to F1-C/VEH.

We tested adult F1 offspring behavior under low- and high-stress conditions. Initial *n*’s, exclusion criteria, and final *n*’s are summarized in Table S1. Male and female data were analyzed separately, since a main effect of sex and/or interactions with the sex variable were observed in all tests (see SI). In the low-stress cohort (Fig. 4a, b), male F1-PRS rats generally showed anxiogenic behavior: they spent less time in the center of the OF (Fig. 4a, left, center) and showed reduced overall exploration in the NOR Test Phase (Fig. 4b, center). Total locomotor activity and center latency in the OF, and object exploration in the Sample Phase of the NOR test were unaffected by maternal PRS or drug treatment. F1 female behavior in the OF was unaffected by maternal PRS or drug treatment (no differences in total locomotor activity, center latency, or center duration, Fig. 4a, right). In the NOR Sample Phase, female F1-PRS/VEH explored more than F1-PRS/NBI and FLX (2 × 3 ANOVA, drug × sex F_2,146_ = 5.007, *p* < 0.01). In the NOR Test Phase, F1-PRS/VEH females exhibited increased exploration compared to F1-C/VEH; maternal NBI and FLX treatment reversed this effect (Fig. 4b, right). Maternal drug treatment had no independent effects in either task. Novel object preference was present in all conditions and was unaffected by PRS or drug treatment in males and females (see SI).

In the high-stress cohort (Fig. 4c–f), maternal PRS led to decreased fear and increased risk-taking behavior in male offspring tested under high-stress conditions; in female offspring, the response was assay-dependent. In the EPM (Fig. 4c, left), male F1-PRS rats exhibited decreased latency to open arms, and this effect was reversed by maternal NBI, but not FLX, treatment (Fig. 4c, center). Male F1-PRS rats exhibited increased frequency (F_1,76_ = 7.1, *p* < 0.01) and a trend towards increased duration in the open arms of the EPM (F_1,76_ = 3.4, *p* = 0.07). Latency to open arms was increased in female F1-PRS/VEH rats, and this effect was absent if maternal PRS was followed by NBI or FLX (Fig. 4c, right). No differences in open arm frequency and duration, or closed arm latency, frequency and duration were found (data not shown).

In the same rats, we measured freezing during delay fear conditioning (Day 1), contextual and cued recall tests (Days 2 and 3, respectively), and cue extinction (Days 3–5). Data from some rats were excluded because of stereotypic behavior, which was not influenced by maternal PRS or drug exposure (see exclusion criteria in Table S1). On Day 1 (Fig. 4d, left), freezing during tone presentation increased gradually in male and female offspring, indicating intact conditioning to tone (repeated-measures ANOVA, F_2,128_ = 208.1, *p* < 0.0001, F_2,156_ = 414, *p* < 0.0001, respectively). Male F1-PRS rats froze less than F1-C controls during the entire tone period and maternal drug treatment did not reverse this effect (Fig. 4d, center). In females, average freezing during the 3 tone presentations, but not the entire tone period, was reduced by maternal PRS, as well as by maternal FLX administration (Fig. 4d, right). On Day 2 (Fig. 4e, left), male F1-PRS rats froze less than F1-C controls (Fig. 4e, center); in females, F1-PRS/FLX rats tended to show the lowest freezing levels overall (Fig. 4e, right). On Day 3 (Fig. 4f, left), we found increased freezing during the average of the first 3 tones compared to the pre-tone period in males (F_1,78_ = 288.6, *p* < 0.001) and females (F_1,85_ = 240.0, *p* < 0.0001), and gradual extinction of fear during the 7 subsequent tones in males (tone, F_4.4,343.2_ = 25.7, *p* < 0.001). Maternal PRS or drug treatment had no effect on freezing during tone recall (tones 1–3) or extinction (tones 4–10) in males (Fig. 4f, center). Female F1-FLX rats froze less than F1-VEH and F1-NBI rats, regardless of maternal PRS, during tone recall and extinction (Fig. 4f, right). No maternal PRS effects were observed on Day 4 or 5 (see SI).

### F2 neonates: basic attributes

Naïve female rats mated with F1-PRS males gained more weight during pregnancy and weighed more than those mated with F1-C males 30 days after parturition (Table S7), although there were no differences in litter size or pup weight (Table S8).

### F2 adults: behavior

**Fig. 5:**
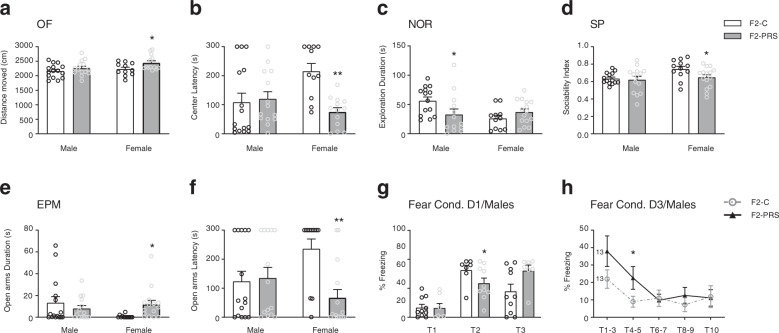
PRS-induced behavioral changes in F2 male and female offspring. **a** F2-PRS females, but not males, exhibit higher locomotion during the first 5 min of the OF test (one-way ANOVA, F_1,22_ = 4.7, *p* < 0.05). **b** Female, but not male, F2-PRS rats show decreased latency to enter the OF center (one-way ANOVA, F_1,22_ = 23.1, *p* < 0.0001). **c** Male, but not female, F2-PRS rats exhibit less exploration in the Test Phase of NOR compared with F2-C males (one-way ANOVA, F_1,25_ = 4.3, *p* < 0.05). **d** Female, but not male, F2-PRS rats demonstrate a lower sociability index (one-way ANOVA, F_1,25_ = 4.5, *p* < 0.05). In EPM (**e, f**), Female F2-PRS rats spend more time in the open arms (**e**; one-way ANOVA, F_1,25_ = 6.9, *p* < 0.05) and enter the open arms sooner (**f**; one-way ANOVA, F_1,25_ = 14.8, *p* < 0.001) than their F2-C controls. No PRS effects are found in male F2 offspring. **g** Fear conditioning, Day 1: PRS induces lower freezing levels in male F2 offspring, particularly during the second tone presentation (**g**; repeated-measures ANOVA, tone × group F_2,30_ = 3.4, *p* < 0.05). **h** PRS has no effect on freezing during the first 3 tones in male F2 offspring. During the 7 subsequent tones, F2-PRS rats extinguish the fear response slower than their F2-C counterparts. In particular, freezing in male F2-PRS offspring tends to be higher during the first extinction tones (tone, F_2.84,68.2_ = 3.2, *p* < 0.05, group F_1,24_ = 3.5, *p* = 0.073). Data presented as individual values, with bars and whiskers representing means and standard errors, respectively (**a–g**) or as means and standard errors (**h**). Post-hoc *p < 0.05, **p < 0.001, relative to F2-C.

Behavioral analysis in adult F2 offspring revealed that in most tasks, grandmaternal PRS affected female, but not male, offspring. In the OF, PRS induced higher locomotion during the first 5 min in F2 female offspring; no differences were found in males (Fig. 5a). Analysis of the latency to enter the center(s) revealed that F2-PRS females entered the center of the OF sooner than their F2-C counterparts. Again, no differences were observed in males (Fig. 5b). No differences were found in males or females in the time spent in the center of the OF (not shown). In the NOR assay, grandmaternal PRS had no effect on exploration time or the latency to approach either object during the Sample Phase (data not shown). In the Test Phase (Fig. 5c), male F2-PRS explored less than F2-C. Notably female F2-C also explored less than male controls. Neither F2-C nor F2-PRS rats showed a preference towards the novel stimulus (not shown). In the SP task, female F2-PRS rats exhibited lower scores in the sociability index compared to F2-C; no differences were found in males (Fig. 5d).

In the EPM, female F2-PRS rats spent more time in the open arms (Fig. 5e), and entered the open arms sooner (Fig. 5f) and more frequently (F_1,25_ = 12.3 *p* < 0.01) compared with their F2-C controls; no differences were found in males. Notably, F2-C females spent significantly less time and showed greater latency to enter the open arms than F2-C males (Fig. 5e, f).

In the fear conditioning and extinction task, male F2-PRS rats exhibited high freezing levels on Day 1 compared with F2-C controls, particularly during the second tone presentation (Fig. 5g). In females, freezing was not affected by grandmaternal PRS (data not shown). Grandmaternal PRS had no effect on freezing to context (Day 2) or during the first 3 tones (Day 3), in either males or females (two-way ANOVAs; not shown). Analysis of the 7 subsequent tones on Day 3 revealed a tendency for slower extinction of the fear response in male F2-PRS rats (Fig. 5h). No significant PRS effects were found in females (not shown). On Day 4 and 5, we found low freezing levels (<15%), and no extinction effects in either males or females (not shown). Rat exclusion details and further analysis details are presented in Table S2 and the SI.

### F1 and F2 adults: mRNA and miRNA expression changes

**Fig. 6:**
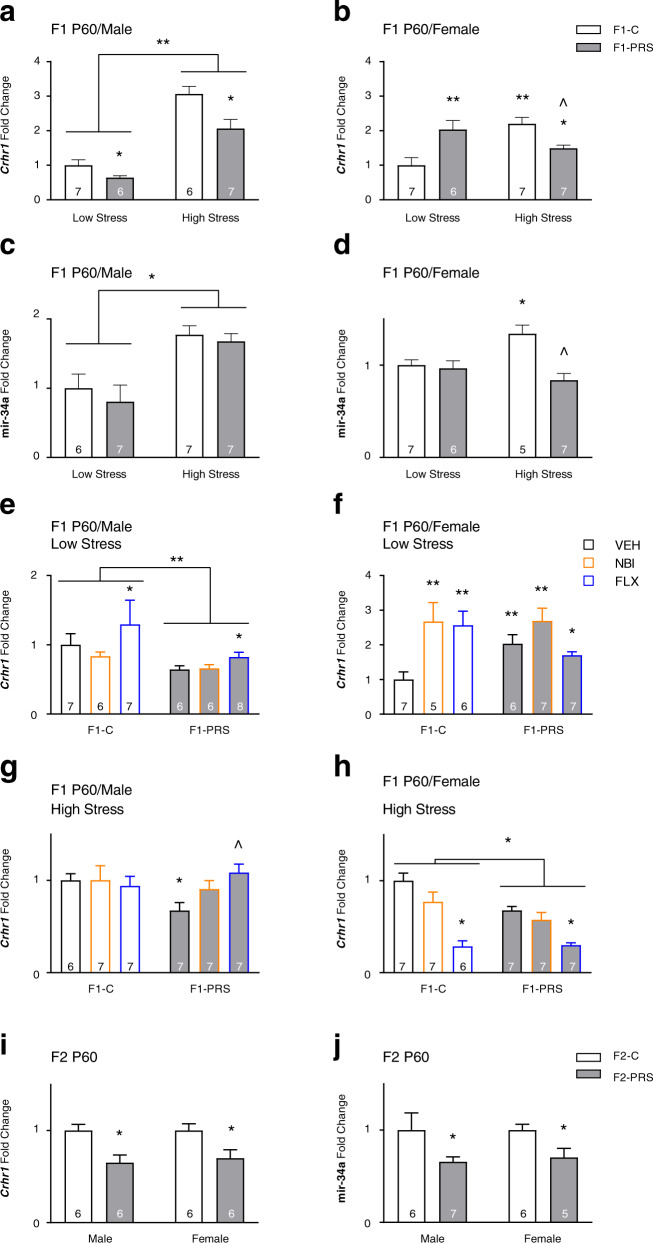
Effects of maternal PRS and drug treatment, and offspring exposure to behavioral stress on *Crhr1* and mir-34a expression in mPFC of adult F1 and F2 offspring. **a** In F1-VEH males, direct exposure to high-stress testing conditions increases, whereas maternal PRS decreases, *Crhr1* expression (2 × 2 ANOVA, cohort F_1,22_ = 117.08, *p* < 0.00001; group F_1,22_ = 15.6, *p* < 0.001). **b** In F1-VEH females, high-stress test exposure increases *Crhr1* expression, whereas maternal PRS increases *Crhr1* expression in low-stress but decreases it in high-stress rats (group × cohort F_1,23_ = 19.2, *p* < 0.001). **c** In F1-VEH males, high-stress test exposure increases mir-34a expression (2 × 2 ANOVA, cohort F_1,23_ = 10.55, *p* < 0.01). **d** In F1-VEH females, high-stress test exposure increases mir-34a expression in F1-C rats, and maternal PRS decreases expression in high-stress rats only (2 × 2 ANOVA, group × cohort F_1,21_ = 7.06, *p* < 0.05). **e** In low-stress F1 males, PRS leads to a decrease in *Crhr1*, and maternal FLX treatment increases *Crhr1* expression (2 × 3 ANOVA, group F_1,34_ = 16.5, *p* < 0.001; drug F_2,34_ = 4.8, *p* < 0.05). **f** In low-stress F1 females, maternal PRS and/or drug treatment increase *Crhr1* expression (2 × 3 ANOVA, group × drug F_2,32_ = 7.9, *p* < 0.01). **g** In high-stress F1 males, maternal PRS decreases *Crhr1* expression, and this is reversed by maternal FLX treatment (group × drug F_2,35_ = 3.1, *p* = 0.059). **h** In high-stress F1 females, maternal PRS and/or FLX treatment decreases *Crhr1* mRNA (group F_1,35_ = 4.9, *p* < 0.05; drug F_2,35_ = 42.2, *p* < 0.001). In F2 offspring, PRS decreases *Crhr1* (**i**) and mir-34a (**j**) expression in male and female offspring (1-way ANOVAs *Crhr1:* M F_1,10_ = 5.43, *p* < 0.05; F F_1,10_ = 5.6, *p* < 0.05; mir-34a: M F_1,11_ = 5.42, *p* < 0.05; F F_1,9_ = 6.3, *p* < 0.05). Data presented as means and standard errors of fold-change. *p < 0.05, **p < 0.001, post-hoc or main effects relative to C-VEH controls. ^*p* < 0.05, post-hoc relative to high-stress F1-VEH or F1-PRS/VEH.

In agreement with our previous observations^28,29^, direct exposure to low- and high-stress testing conditions interacted with maternal stress exposure in its impact on F1 *Crhr1* expression (Fig. 6a, b), with different effects in male and female rats. In males, high-stress testing conditions increased, but maternal PRS decreased, *Crhr1* mRNA expression (Fig. 6a). In females, high-stress testing increased *Crhr1* mRNA. Maternal PRS increased *Crhr1* expression in low-stress, but decreased it in high-stress females (Fig. 6b). We asked whether increased *Crhr1* expression would be accompanied by decreased mir-34a expression, as observed in germline cells and neonates, but changes in mir-34a seemed to vary independently of *Crhr1* in adults. Mir-34a expression was increased in males exposed to high-stress conditions, regardless of maternal PRS (Fig. 6c). In females, exposure to high-stress conditions increased mir-34a expression in offspring of Control dams (Fig. 6d), and maternal PRS decreased expression in high-stress, but not in low-stress, offspring.

Maternal drug treatment reversed the impact of maternal PRS on *Crhr1* expression in male offspring exposed to high-stress, but not low-stress, conditions, and FLX had independent effects on low-stress offspring (Fig. 6e, g). In female F1 offspring, maternal PRS or drug treatment led to increased expression under low-stress conditions (Fig. 6f). Under high-stress conditions, maternal PRS or FLX decreased the *Crhr1* expression whereas NBI had no effect (Fig. 6h). Examining the impact of maternal NBI or FLX treatment on serum CORT in control and PRS-exposed females and their offspring, we found that maternal NBI treatment, which reversed the impact of PRS on serum CORT in F0, also reversed the 3.5-fold PRS-induced increase in CORT observed in behaviorally naïve F1 offspring^28^, whereas FLX administration had the opposite effect and increased CORT by >8 fold (Table S9).

In F2, we assessed rats that underwent a high-stress testing battery. As in the F1 high-stress cohort, PRS decreased *Crhr1* mRNA expression (Fig. 6i). It also significantly decreased mir-34a expression in both male and female F2 offspring (Fig. 6j).

We examined maternal PRS-induced fluctuations in the expression of *Crhr2* in adult F1 offspring tested in low-stress and high-stress conditions (Fig. S4). We found that *Crhr1* and *Crhr2* were negatively correlated high-stress F1-PRS rats (*n* = 12, *r* = −.842, *p* = 0.001); there was no correlation in low-stress rats or in F1-C offspring. In male F1 offspring, *Crhr2* expression was increased by both maternal PRS and exposure to high-stress conditions, but was normalized to control levels in high-stress/F1-PRS offspring (Fig. S4a). In female offspring, the opposite pattern was observed: *Crhr2* expression was decreased by both maternal PRS and high-stress testing conditions, but high-stress/F1-PRS females showed normal *Crhr2* expression (Fig. S4b). Drug treatment normalized aberrations in *Crhr2* expression in low-stress males (Fig. S4c) and females (Fig. S4d), as well as in high-stress males (Fig. S4e). In high-stress females, maternal drug treatment had no effect (Fig. S4f). In F2 offspring (exposed to high-stress conditions), *Crhr2* was decreased in male, but not in female, offspring (Fig. S5).

## Discussion

Chronic, unpredictable stress in adolescence, prior to gestation, leaves molecular and behavioral footprints in subsequent generations. Here, we demonstrate for the first time that exposure of female rats to PRS induces changes in blood and oocyte miRNA expression, and similar changes in miRNA expression in the PFC of neonate F1 and F2 offspring. Furthermore, we find that some of the effects of PRS in offspring can be reversed by maternal pharmacological interventions. Finally, we show that FLX treatment in adolescence impacts on reproductive health and offspring stress susceptibility.

Four days after stress exposure, we observe an increase in *Crhr1* mRNA in PRS-exposed dams, in line with our previous findings^28,29^. In parallel, the expression of mir-34a and mir-34c, which target *Crhr1* and block its transcription^32,37,51^, is reduced in oocytes of PRS rats while the expression of non-*Crhr1*-targeting miRNAs is unaltered (Figs. 2g and S3). The mir-34 family of microRNA molecules are associated with the stress response^52^ as well as with longevity and brain aging^53^. Specifically, alterations in mir-34 are associated with resilience under stress conditions^51^. This is the first report of stress-induced alterations in rat oocyte miRNA expression. miRNA expression changes have been detected in mouse sperm^20,21,26,54^, and were suggested to provide a mechanism for epigenetic germline inheritance in mammals^55–57^. Oocyte miRNAs may play a similar role, and transmit not only genomic but also maternal epigenomic information across generations^27^. Notably, the increase in oocytic *Crhr1* is transient, while the decrease in mir-34 remains low at the time of mating. The mechanism underlying the translation of behavioral stress to altered expression of germline miRNA molecules is unknown, and may involve HPA axis activation^58–61^ affecting blood–germline cell communications^20,62–64^. In support of this possibility, our previous studies point to increased CORT levels, and our present findings show increased *Crhr1* mRNA and decreased miR-34a in blood of stress-exposed F0 females (Fig. 2d, h). Notably, *Crhr2* (which is also targeted by mir-34) was not detected in blood or oocytes.

Changes in mir-34a and mir-34c expression in oocytes of stress-exposed dams may be directly or indirectly responsible for the reduction in *Crhr1* mRNA observed in the PFC of their neonate F1 and F2 offspring (Fig. 3a, b). Previous studies have shown that mir-34c reduces the responsiveness of cells to CRF in vitro^32^. Thus, changes in germline mir-34 expression could alter sensitivity to HPA axis signals *in utero* and impact cortical development in F1. Alternatively, reduced *Crhr1* at birth could stem from abnormal *in utero* cortical development. Future studies should examine whether miRNA expression patterns are also altered in sperm of F1-PRS males, which in the present study were mated with naïve females to produce F2 offspring. Such alterations could account for *Crhr1* expression abnormalities in the brain of neonate F2 offspring.

The reduction in cortical *Crhr1* expression in F1 and F2 neonate brain was accompanied by an increase in mir-34a expression. This increase could substantially hinder cortical maturation; overexpression of mir-34a in cortical neurons was found to increase cellular vulnerability^65^. In our model, overexpression could lead to mal-programming of the HPA axis and account for our previously reported abnormalities in adult PFC morphology in F1-PRS rats^30^. It should be noted that an inverse pattern of changes (i.e., increased *Crhr1* and decreased mir-34a, similar to patterns observed in oocytes and blood) was observed in neonate AMY in F1 and F2; this observation supports previous evidence for different developmental patterns of the HPA axis in PFC and AMY^66,67^.

In contrast with neonates, stress-induced changes in *Crhr1* mRNA in brain of F0, F1, and F2 adult animals of all three generations were not paralleled by mir-34a expression changes (Figs. 2 and 6). Possibly, stress-induced changes in miRNA expression could be quick and transient, returning to normal levels by P56. Alternatively, changes in mir-34a and *Crhr1* in blood and oocytes as well as in neonate brain may be a specific marker of the inter-generational transfer of stress effects, and not stress per se. Generally speaking, phenotypes observed in adult offspring could be the consequence of stress-induced alterations in maternal care. Notably, such alterations are less likely to account for changes in F2, since F2 offspring were derived from male F1 offspring and naïve females.

Our behavioral assays show that male F1-PRS rats showed decreased center exploration in the OF and reduced novelty exploration in the NOR task (Fig. 4a, b), along with increased exploration of the EPM open arms and less freezing during acquisition of the fear response (Fig. 4c–f). These phenotypes are consistent with the idea that maternal PRS induces in male offspring a more ‘cautious’ phenotype under benign circumstances, but ‘inoculates’ them against acute stress or perceived danger. This is in line with previous studies in humans and animals, which show that exposure to stress in early life or adolescence can result in stress susceptibility and also in resilience, depending on, e.g., type of stressor or offspring sex^6,7,29,67–70^. Further investigations into the advantages and disadvantages of parental stress in various offspring environments are required for in-depth understanding of stress susceptibility and resilience.

In agreement with our previous studies, *Crhr1* expression in adult mPFC depends on maternal as well as offspring exposure to stress (Fig. 6). *Crhr1* expression was higher in offspring exposed to stressful testing conditions compared to benign behavioral tasks. Maternal exposure to stress, however, affected offspring in a sex-dependent manner, decreasing *Crhr1* in males and increasing it in females (Fig. 6). In parallel, maternal PRS mitigated anxiogenic behavior in males, but exacerbated it in females (Fig. 4c–f). Interestingly, expression of *Crhr2*, which is not targeted by mir-34a or mir-34c, was negatively correlated with *Crhr1* expression in F1-PRS offspring and was affected by maternal PRS, sometimes in a pattern opposite to what we observed with *Crhr1* (e.g., Figs. 6b and S4b). However, changes in *Crhr2* were not paralleled by behavioral alterations. Furthermore, *Crhr2* levels in F0 were unaltered by stress, although this effect should be examined at additional time points. *Crhr1* expression may thus be more closely associated with changes in anxiogenic behavior, and expression of *Crhr1* and *Crhr2* in offspring is likely to be mediated by different mechanisms.

Some of the behavioral and molecular findings in adult rats agree with our previous findings^28,29,49,71^, while others differ. This may be due to differences in the order and identity of the behavioral assays, which could have affected behavioral and, well as, gene expression profiles. For example, the ‘low-stress’ behavioral cohort in the present study included the OF, NOR, and SP tests, while in our previous study ‘low-stress’ rats were tested in OF only. The NOR and SP tasks involve novelty exposure, which impacts gene expression patterns in the mPFC^72^ and engages the HPA axis^73^; this may explain differences in cortical *Crhr1* expression patterns between the current study and our previous investigations. Similarly, differences in fear acquisition between our present and past findings may be accounted for by previous exposure to a fear-eliciting environment: in the present study, rats were first exposed to the high-stress EPM assay, whereas previously they were tested in the fear conditioning assay alone. These differences highlight the complex interactions between parental and direct exposure to environmental factors, e.g., novelty or stress, and merit further investigation. Sex differences in both F1 and F2 offspring are in line with our previous experiments and a plethora of other studies (e.g.,^74^), and could result from differential interference of stress with sex hormone signaling, or from sex-dependent differences in epigenetic regulation^75^.

CRHR1 antagonists were previously shown to reverse the sequelae of early developmental or adult stress^76,77^, CRF administration^78^, and mir-34 knockout^37^. Here, maternal post-PRS subchronic treatment with the CRHR1 antagonist NBI reverses PRS-induced changes in *Crhr1* expression in neonate offspring, as well as abnormalities in serum CORT and behavior in adult progeny (Figs. 3, 4c–f, and Table S9). This is the first demonstration of combined stress and drug effects in adult offspring of exposed rats, and further supports the role played by *Crhr1* elevation in the transmission of stress effects.

Surprisingly, subchronic administration of FLX to adolescent females, prior to gestation, increases pup mortality and reduces offspring weight from birth to early adulthood, particularly when followed by stress exposure. Administration of high-dose FLX and other SSRIs during gestation and lactation was previously demonstrated to increase neonatal mortality and decrease birth weight in rodents^79–85^. In our study, FLX was administered pre-gestationally and at a relatively low dose. FLX is commonly administered during adolescence, a time period of heightened stress sensitivity^86^. However, the impact of adolescent pre-gestational FLX use on neonatal viability and early brain development has scarcely been explored^87^.

In adult offspring, maternal FLX treatment exacerbated the effect of maternal PRS on serum CORT, but had some beneficial effects on behavior in offspring of stress-naïve as well as PRS rats (Fig. 4 and Table S9). The latter findings are in agreement with prior rodent literature, where FLX reversed stress-induced anxiogenic and depressive symptoms as well as memory impairments in offspring^88,89^. The mechanism/s underlying the effects of FLX in neonate and adult offspring remain to be determined, and may involve an ongoing effect of FLX and its active metabolite, norfluoxetine, on the developing fetus despite discontinuation of FLX a week prior to mating^90,91^. FLX could also affect neonate and adult offspring phenotypes by its indirect effects on the blood–brain barrier^92^ and the HPA axis^93–99^. Another interesting possibility is that FLX treatment affects the quality of maternal care^100^; this possibility should be examined in future studies.

As in our previous study^28^, we observed *Crhr1*, CORT, and behavior changes in adult PRS F2 offspring. Some behavioral effects in the present study were similar in F1 and F2, e.g., NOR exploration times decreased in both F1 and F2 males (Figs. 4b and 5c) and EPM abnormalities in paternally derived F2-PRS females were similar to those found in F1-PRS males (Figs. 4c and 5f). A comparison of the current study with our previous investigation^28^, where an identical experimental design was used but F2 offspring were derived from F1 females, reveals that maternal and paternal transmission produce different behavioral and molecular phenotypes in offspring. In general, transmission via the paternal lineage leads to behavioral alterations in female offspring, whereas transmission via the maternal lineage affects offspring of both sexes.

Interestingly, naïve females mated with PRS F1 males gained significantly more weight during pregnancy. This may reflect emotional transfer effects^101,102^, and points to an altered *in utero* environment affecting F2 phenotypes. Germline cells of F1 offspring, as well as maternal behavior in F1, should also be examined to clarify the relative roles of social and epigenetic mechanisms in transmission.

**Fig. 7:**
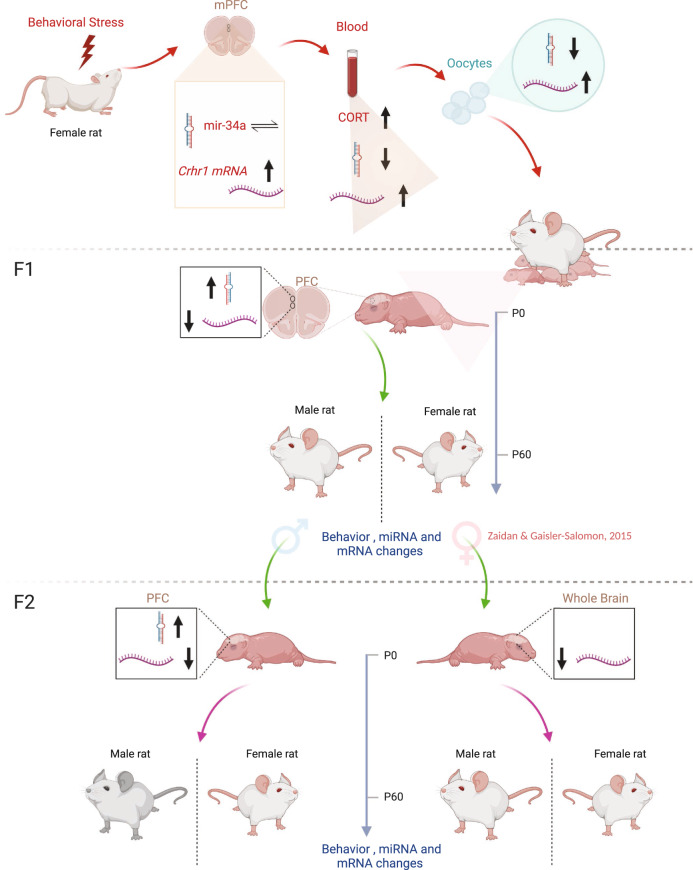
Schematic summary of findings in present study and previous investigations of stress transfer in this model. A potential mechanism for the inter- and trans-generational transfer of stress effects may involve stress-induced *Crhr1* elevations in F0 brain, blood and oocytes; changes in mir-34 expression in blood and oocytes may be specific markers for information transfer. Similar fluctuations in *Crhr1* and mir-34a are observed in PFC (and AMY, not shown) of neonate F1 and F2 progeny. Transfer via the maternal or paternal line affects male and female F2 progeny differently.

In sum, our findings point to epigenetic mechanisms as a putative mediator of stress transmission across generations (see summary Fig. 7). Clearly, these mechanisms may interact with social factors, i.e., maternal care, which were also shown to impact similar stress-related pathways^103,104^. This interaction should be more extensively investigated in future studies. Furthermore, the findings of the present investigation indicate that pharmacological intervention may be effective in reversing some of the effects of stress across generations, while having its own impact on some measures. Finally, this study highlights the importance of studying stress transmission, susceptibility, and resilience in both genders, since the impact of adversity and mechanisms of transmission differ significantly between males and females.

## Supplementary information

Supplemental Methods and Results.

Supplemental Tables.
