# Supplementary material for: Pre-reproductive stress in adolescent female rats alters oocyte microRNA expression and offspring phenotypes: pharmacological interventions and putative mechanisms
Source: Transl Psychiatry. 2021 Feb 5;11:113. doi: 10.1038/s41398-021-01220-1 (PMC7865076; doi:10.1038/s41398-021-01220-1)
Supplement: Supplementary file 1 — Supplemental Methods and Results. [file 41398_2021_1220_MOESM1_ESM.docx]

**Pre-reproductive stress in adolescent female rats alters oocyte microRNA expression and offspring phenotypes: pharmacological interventions and putative mechanisms**

Hiba Zaidan^1^, PhD; Dalia Galiani^2^, M.Sc & Inna Gaisler-Salomon^1*^, PhD

^1^Department of Psychology, University of Haifa, Haifa, Israel.

^2^Department of Biological Regulation, Weizmann Institute of Science, Rehovot, Israel.

**Supplementary Methods and Results**

## **Animals**

Virgin adolescent female Sprague-Dawley rats and adult males used for mating were purchased from Envigo (Jerusalem). Housing conditions (except during the stress procedure) included wood-flake bedding, ad lib food and water, 12h artificial lighting during the day (07-19h), and temperature maintained at 22±2oC. Rats were minimally handled except for weekly weighing, and soiled bedding was periodically only partially replaced, without removing the rats, so that home-cage conditions were minimally disrupted.

Animals were randomly distributed across groups (see Experimental procedure below) and were handled in accordance with the NIH guidelines for the Care and Use of Laboratory Animals, 8th edition[^1^](#_ENREF_1).

## **Experimental Procedure**

The experimental timeline is outlined in **Fig 1**. One hundred and four adolescent (P45) female rats were group housed (4-6 rats per cage) in 56x35x19cm cages. Cages were randomly divided into control (C) and PRS groups (n: C 53, PRS 51). PRS rats underwent a 7-day chronic unpredictable stress (CUS) procedure as previously described^2-6^. Following the CUS procedure, the rats were returned to their original cages. Twenty-four hours later (P53), females from C and PRS groups were divided into 3 cohorts. Rats in cohort 1 underwent a 2-day ovulation induction procedure (performed as previously described^6, 7^) starting on P53 (n: C 7, PRS 9). On P56 (4 days after the end of the CUS procedure) mature oocytes were counted and collected, and brain was extracted. Oocyte samples were used for mRNA analysis, miRNA analysis, or both. In cohort 2, rats underwent a 2-day ovulation induction procedure, and mature oocytes were counted and collected during P66-73 (equivalent to the breeding phase). Trunk blood was also collected. Oocytes and blood samples were used for mRNA and miRNA analysis. Rats in cohort 3 were randomly injected intraperitoneally (IP) with either vehicle (VEH), the selective CRHR1 antagonist NBI 27914 (NBI; 5 mg/ml; 5 days) or the selective serotonin reuptake inhibitor fluoxetine (FLX; 5 mg/kg, injection volume 0.5ml) for 7 consecutive days, starting on the day following the end of the CUS procedure) n=7-10/group, see **Table S4**). During P66-73 (equivalent to the breeding period), several randomly selected rats from the VEH groups were sacrificed for *Crhr1* mRNA and miRNA expression analysis (n=5-10/group). The remaining females from each group (n=10-11/group) were mated with behaviorally naïve adult male rats and were removed from the mating cage 7 days later. Female rats were returned to their home cage; pregnancy was verified by weekly weighing. Each pregnant rat was moved to a 37x30x19cm cage 7 days prior to parturition. Stress or drug treatment had no effect on pregnancy rates (70-90%) or on oocyte viability (see **Table S4**).

On P0 (up to 24hr after birth), the litters were culled up to 10 pups (approximately equal numbers of males and females). The number of stillborn in each litter was assessed (see **Table S4**). A subset of F1 male and female neonate pups randomly selected from all litters was sacrificed for mRNA and/or miRNA expression analysis (n=6-9/group). Remaining pups remained in their home cages undisturbed until P30, then weaned and raised in same-sex, same-condition groups of 4–6. Randomly selected dams from each group were sacrificed after weaning; blood samples were collected for assessment of CORT levels (n=4-5/group).

On P60, a subset of randomly-selected behaviorally-naïve F1 males and females from all groups and litters were sacrificed. Blood samples were collected for assessment of CORT levels in selected conditions (FLX females and NBI males and females; n=4-8/group, see **Table S9**). The remaining animals were randomly assigned into 2 cohorts and underwent behavioral testing under low- and high-stress conditions (see details below and **Table S1** for initial/final n’s). Brains were removed 1 day after the end of the behavioral assessment, and mPFC was extracted for gene and miRNA expression analysis (n=5-7/group).

Five behaviorally-naïve adult male F1-C and 6 F1-PRS offspring of VEH-treated dams were each mated with 2 naïve adult female rats. Mating, pregnancy, weighing and weaning of F2 pups were conducted as described above for F1.

On P0, F2 pups were sacrificed for mRNA and miRNA expression analysis (n=7-9/group). Remaining pups were raised undisturbed until P30, then weaned and raised in same-sex, same-condition groups of 4–6. Adult (P60) F2-C and F2-PRS males and females rats underwent behavioral testing (n=13-15/group; see **Table S2** for details) and were sacrificed 24 h later. mPFC was extracted for gene expression analysis (n=5-7/group).

## **Drugs**

We relied on previous work with NBI^8-10^ and FLX^11-14^ to determine dose and administration regimes. NBI (Sigma- Aldrich, St. Louis, MO) and FLX (Sigma- Aldrich) were dissolved in dimethyl sulfoxide (DMSO) and diluted with 0.9% saline to a concentration of 5 mg/ml with a final DMSO concentration less than 0.1%. Drugs were administered i.p. at a volume of 2

ml/kg for 5 days (NBI), or 7 days (FLX). NBI-injected rats received 2 additional days of VEH injections so that the total injection period for all groups was 7 days. The VEH group was injected with 0.1% DMSO in saline.

**Behavioral Tests**

F0: Dams were tested for general locomotor abnormalities and novelty-induced anxiogenic behavior in the open field (OF) for 5 min. Since we wanted to minimize exposure to stressful conditions in C dams, this 5-min procedure was the only behavior assessed. F1: Male and female adult progeny were randomly divided into 2 testing cohorts, to enable comparison between low- and high-stress exposure. Cohort 1 (‘low stress’) was tested in the OF followed 24h later by Novel Object Recognition (NOR). Cohort 2 (‘high stress’) was tested in the Elevated Plus Maze (EPM) followed 24h later by the fear conditioning and extinction test. Previous studies show that anxiogenic behavior generated by a distinct cue (i.e., a footshock or an elevated pedestal) may differ from continuous anxiogenic behavior e.g. in the open field arena, and may be mediated by different brain regions^15^, and our own previous studies indicate that the molecular sequalae of low- and high-stress are different^5, 6^. F2: Male and female adult progeny were tested in the OF, NOR and Social Preference (SP). Each test was separated from the next by 24 hrs. Two weeks later, rats were tested in the EPM followed 24 h later by fear conditioning and extinction test. Male and female F1 and F2 rats were tested on separate days.

Open Field (OF)

Rats were tested in the OF test to assess locomotor, exploratory and anxiogenic behavior^16^ as previously described^5^. Rats were placed in a dimly-lit open field arena (58x58x20h cm) for 5 min in dams or 15 min to offspring. Total locomotor activity and time spent in the center were assessed with EthoVision XT 10.0 (Noldus Information Technology, Wageningen, Netherlands).

## Novel Object Recognition (NOR)

The NOR test is commonly used to assess object recognition and working memory^17, 18^. Testing was conducted in the same arena used for OF for 5 min using dim light. The objects were children’s plastic building blocks of comparable height but of different shape (e.g., cube or pyramid) and hue (e.g., bright yellow, dark blue). Objects were placed 12 cm away from two diagonal corners and fixed to the chamber floor with double-sided adhesive tape to prevent them from being moved.

Twenty-four hours prior to behavioral testing, each rat was given a 10 min exploration period in the box to ensure habituation to the empty apparatus and test room environment. The test was conducted in two phases, a Sample and a retention Test phase. During the Sample Phase, two objects (A and B) were placed in the experimental chamber. Each rat was placed in the center of the arena and was allowed to explore the two objects freely for a period of 5 min. Rats were then returned to their home cage for a 5 min inter-trial interval (ITI). During this time, the arena and the objects were wiped clean with 70% ethanol, and one of the objects was replaced with a novel object. The location of the novel object in the retention trial (left or right) was counterbalanced between groups. After the ITI, rats were returned to the box for the Retention Test Phase, during which they were allowed to explore the familiar and novel object in the test box for 5 min. Object exploration was defined as the rats sniffing, licking or touching the objects with forepaws while sniffing but not by leaning against, turning around, standing or sitting on the objects^19^. Exploration was assessed using EthoVision XT 10.0. We analyzed the exploration time (s) of each object during both sessions of the NOR test. In the Test Phase, we also recorded the latency and frequency of approach for each object, and calculated a novelty ratio (novel/ total exploration time) and the frequency ratio (frequency of approaching the novel object/total frequency). Rats that did not complete 30 seconds of total exploration time in the Sample Phase were excluded from the analysis of the novelty ratio; all data was included for the analysis of exploration in both stages.

## Social Preference (SP)

The social preference test is commonly used to assess rodent sociability, and has been conducted as previously described^20^. Briefly, the arena (40lx70wx30h cm) was divided into 2 unequal compartments by a transparent perforated Plexiglas panel, allowing for intact visual and olfactory cues. The experiment rat and an unfamiliar con-specific partner rat of the same sex and age were placed in different sessions in the larger and smaller compartment, respectively, for a 5-min habituation period. Twenty-four hours later, the partner rat was placed in the smaller compartment, and an unfamiliar object (plastic, 5x5x8h, 11x8x8h cm) was placed in the larger compartment, 10 cm diagonally from the corner of the arena. One minute later the experiment rat was placed in the arena for 5 minutes. Several objects and partner rats were used throughout the experiment, and were counterbalanced between groups. Time spent exploring the partner rat and the object was measured using EthoVision XT10.0. Rats that did not complete 30 seconds of total exploration time were excluded from the experiment.

## Elevated Plus Maze (EPM)

The EPM, a ‘+’-shaped maze positioned ~60cm above the floor with crossing ‘open’ and ‘closed’ (walled) runways, is commonly used to assess anxiogenic behavior in rodents^21^. Testing was performed as previously described^5, 6^. Rats were brought to the test room in pairs, remained in their cages for a 5 min habituation period, and were then individually placed in the center of the EPM for 5 min. Exploration time in the open and closed arms (s) and arm entry latency (s) and frequency (arm entries) were assessed^22-24^ using EthoVision XT 10.0.

## Fear conditioning

A delay fear conditioning procedure was used to assess Pavlovian fear learning and retrieval, i.e., rats’ ability to associate a neutral conditioned stimulus (CS; tone) and a particular context with an aversive unconditioned stimulus (US; mild foot-shock;^25^). AMY-PFC circuitry plays a key role in the acquisition, consolidation and retrieval of fear memory, as well extinction of fear^26-28^. Rats were trained and tested in the conditioning chambers (41×27×27h cm; Coulbourn Instruments, Whitehall, PA) using a 5-day procedure conducted as previously described with slight modifications^6, 29^. Freezing was assessed using FreezeFrame software (ActiMetrics, Wilmette, Illinois), which assesses freezing by measuring changes in pixel intensity between successive video frames. On Day 1 (Acquisition), rats were allowed 2 min of exploration and then presented with 3 tone-shock pairings consisting of a 30 s tone (2000 Hz, 80 dB) co-terminating with a 0.5 s (0.8 mA) foot shock, with an inter-stimulus-interval (ISI) of 2 min. Freezing was calculated for each tone and during the entire tone period (from the beginning of the first tone to the end of the session). On Day 2 (Context Test), rats were placed in the same context as on Day 1 for 9.5 min without tone or shock presentation. Freezing during the entire test minus the first minute was calculated. On Day 3 (Tone Test and Extinction), rats were placed in a different context, which consisted of altered visual and tactile cues, and after a 2 min exploration period were presented with 10 tones (30 s, 2000 Hz, 80 dB) separated by a 2 min inter-stimulus interval. No shocks were administered. Freezing was calculated for the first 3 tones as average (tone test) and each of the subsequent 7 tones separately. On Day 4 and 5 (Extinction), rats were placed in the same context as Day 3 and underwent the same protocol. Freezing during each pair of tones was analyzed.

## **Blood Collection for CORT and mRNA/miRNA Quantification**

CORT is the most abundant circulating steroid secreted by rodents and is considered to be an indicator of HPA axis activity^30^. Blood collection and quantification were carried out in the morning, as previously described^5^. Briefly, rats were decapitated, and trunk blood was collected promptly. Serum was prepared by centrifuging blood samples for 10 min at 2000 rpm (4 °C). Fifty μl of serum were collected and CORT levels were quantified using a commercially available enzyme-linked immuno-absorbent assay (ELISA, IBL International GmbH, Hamburg, Germany). The intra-assay coefficient of variation was 2.92%. All comparisons were made between samples run on the same plate. For assessment of gene and miRNA expression, trunk blood was collected and stored in Vacuette K2EDTA tubes (Greiner Bio-one, Austria) at -80 °C. RNA was extracted using Monarch Total RNA Miniprep Kit (New England Biolabs, USA) according to the manufacturer’s instructions.

## **Quantitative real-time PCR (qRT-PCR) for assessment of RNA/miRNA expression**

Rats were sacrificed by decapitation and bilateral samples from PFC (neonates) or mPFC (adults; consisting of prelimbic and infralimbic regions) and AMY (neonates: entire AMY; adults: central and basolateral nuclei) were removed by cryostat^31, 32^ using 0.5 or 1.0 mm punches. Oocytes were extracted from treated animals as previously described^6, 7, 33^. Briefly, 24 hours following the stress procedure, females from each group underwent ovulation induction by intraperitoneal injecting of 40 IU pregnant mare's serum gonadotropin (Sigma-Aldrich, St Louis, MO). in 300 μl of phosphate-buffered saline (PBS), followed 48 h later by 40 IU of human chorionic gonadotropin (Sigma-Aldrich) in 300 μl ml PBS. Rats were sacrificed 24 hours later by decapitation, oviducts were excised and flushed into medium consisting of Leibovitz's L-15 tissue culture medium (Gibco, Grand Island, NY) containing penicillin (100 IU/ml, Gibco) and streptomycin (100 lag/ml, Gibco) and hyaluronidase enzyme (1 mg/ml, Sigma, 10 min, 37°C). Oocytes were counted and collected.

All samples were immediately placed on dry ice and kept at -80^0^C until further processing. mRNA/miRNA extraction, cDNA preparation and quantitative real-time PCR (qRT-PCR) were performed as previously described^6, 34^. Briefly, oocytes were homogenized in 500 μl TRIzol Reagent (Invitrogen, Carlsbad, CA) and 10 μl glycogen (Sigma-Aldrich). Dissected brain regions were homogenized in 300 µL of TRIzol, then suspended in a total of 0.5 ml TRIzol. After adding 200 μl chloroform to allow phase separation by centrifugation (14,000 rpm, 10', 4 °C), 250 μl ispropanol (Sigma-Aldrich) was added to the aqueous phase. After a night in −20oC, the RNA was precipitated by centrifugation (14,000 rpm, 15'). The pellet was washed in 500 μl cold 100% ethanol and centrifuged (7,600 rpm, 10'), then washed in 500 μl cold 75% ethanol and centrifuged again and then dried. RNA was diluted in 25 µl water and quantities were determined using a Nanodrop 2000 spectrophotometer (Thermo Scientific, Wilmington, DE, USA). The 260:280 nm absorbance ratio was measured to assess RNA quality; samples were excluded if the ratio was outside the range of 1.7-2.0, or if RNA concentration was too low. DNA digestion was carried out with 1 μL DNase (Fermentas), incubated at 37 °C for 30 minutes, and then deactivated at 65 °C for 10 minutes. Reverse transcription was carried out using qScript cDNA Synthesis Kit (Quanta Biosciences, Gaithersburg, USA) following the manufacturer’s protocol. cDNA was amplified in a 10 µL reaction (0.6 µL primers (10µM), 5 µL PerfeCTA SYBR green (Quanta Biosciences, Gaithersburg, USA), 2 µL cDNA (diluted 1:9 with H2O) and 2.4 µL ddH2O), by a Step One RT-PCR machine (Applied Biosystems, Carlsbad, CA), using the following PCR conditions: (95°C for 20 sec Holding stage, followed by 40 cycles (95°C for 3 sec, 60° C for 30 sec). Primers (see **Table S3**) were designed using Primer3 software^35^. Fold-change values were calculated using the ddCt method^36^ relative to the housekeeping gene (HPG) hypoxanthine phosphoribosyl transferase (HPRT) or 18s (oocytes). dCt values for HKGs were similar between groups (<.5 Ct, t-tests: all *p*’s>0.1).

The microRNA databases and target prediction tools TargetScan ([www.targetscan.org](http://www.targetscan.org))^37^ and microRNA.org ([www.microrna.org](http://www.microrna.org))^38, 39^ were used to identify potential miRNA targeting the *Crhr1* sequence, highly conserved in rats, mice and humans. A pilot study was preformed to verify the expression of the selected miRNAs in rat brain and oocytes.

miRNA expression analysis was carried out as described^40, 41^. Briefly, reverse transcription reactions were performed using gene-specific primers and high-capacity cDNA RT kit (Applied Biosystems, Foster City, CA, USA; 20 ng RNA fractions) or the qScript microRNA cDNA Synthesis Kit (Quanta Biosciences, Gaithersburg, USA). miR-34a-5p (assay ID: 000426), miR-34c-5p (assay ID: 000428), miR-382-5p (assay ID: 000572) and U6-snRNA (assay ID: 001973) were measured using the TaqMan miRNA kit (Applied Biosystems) according to the manufacturer's instructions. miR-34a-5p, mir-382-5p, mir-34c-5p, mir-137-5p, mir-137-3p, mir-203-5p, mir-203-3p, mir-493-5p, mir-493-3p expression were assessed using SYBR Green qRT-PCR amplification (5 ng total RNA in 20 μl total reaction volume) using specific primers (0.4 μl each, Quanta Biosciences, Gaithersburg, USA) according to manufacturer’s instructions. RT reactions were carried out using a Step One real-time PCR system (Applied Biosystems). Fold-change values were calculated using the ddCt method relative to the HPGs U6-snRNA or RNU6.

## **Statistical analyses**

Data were analyzed with SPSS 23 Statistics software (IBM, Chicago, Illinois). GraphPad Prism 7.0 (GraphPad Software, San Diego, CA), FunRich 3.1.3^42^ and BioRender (<https://biorender.com/>) were used to create figures. Student’s t-test, analysis of variance (ANOVA), repeated-measures ANOVA and multivariate analysis of variance (MANOVA), and Pearson correlation coefficients were used to analyze behavioral and gene expression data. In light of our previous findings and since present data also pointed to sex differences in behavior and neuroendocrine function, data were analyzed separately for males and females. For repeated measures ANOVA, the Greenhouse-Geisser correction was applied when the assumption of sphericity was violated. The Least Significant Difference (LSD) test was used for post-hoc comparisons when interactions were significant. The Chi Square test of independence was performed to examine the relation between dam exposure to stress and/or drug treatment and pup mortality odds. A litter where mortality occurred was scored as a ‘1’ whereas no mortality was scored as ‘0’. Outliers were defined as two or more standard deviations from the mean and removed from the analysis. Data excluded from the analysis, for either technical or statistical reasons, are detailed in **Table S1** (F1), and **Table S2** (F2). Significance level was set at *p*<.05. Results that approach significance were defined as .05≤*p*≤.075.

**SI Figures/Figure Legends**

**
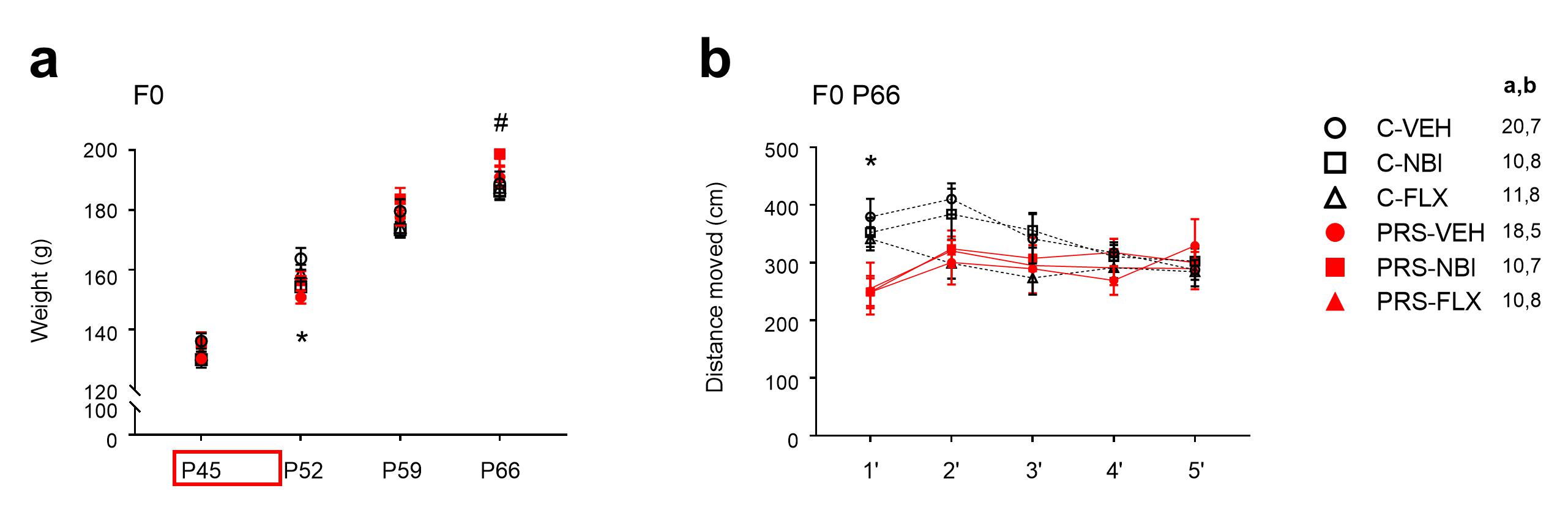
Fig S1.**

**Fig S1.** **PRS- and drug - induced weight, locomotor activity changes in control (C) and pre-reproductive stress (PRS)-exposed F0 females.** (**a**) There were no differences in weight prior to stress exposure (P45). PRS in adolescence induces short-term weight loss (P52) and long-term weight gain (P66) in exposed females, regardless of drug treatment (repeated-measures ANOVA, time x group F_1.5,107.6_=22.2, *p*<0.0001). Overall, C rats gained 54.3±1.4 g between P45 and P66, while PRS-exposed rats gained 61.0±1.8 g. (**b**) PRS females show decreased locomotor activity during the 1st min of the OF test, regardless of drug treatment (repeated-measures ANOVA, time x group F_4,148_=6.1, *p*<0.001). #*p*<0.075. **p*<0.05, relative to C.


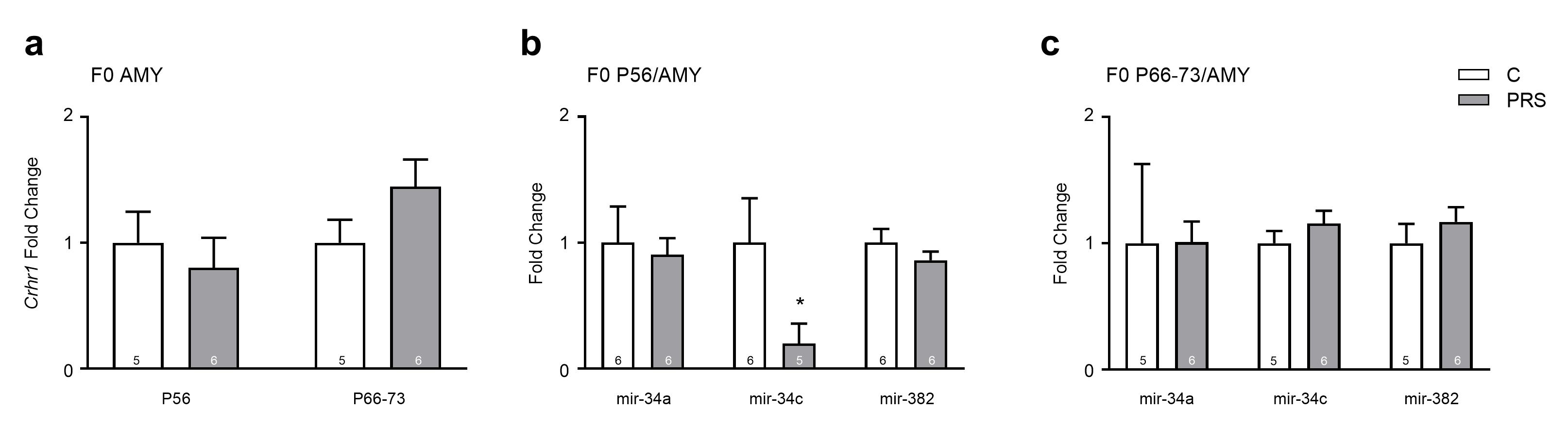
**Fig S2.**

**Fig S2.** **PRS -induced changes in *Crhr1* mRNA and miRNA expression in F0 AMY.** (**a**) No changes in *Crhr1* mRNA expression were observed in the AMY on either P56 or P66-73. (**b**) PRS exposure decreased mir-34c expression (1-Way ANOVA, F_1,11_=5.8, *p*<0.05), with no change in mir-34a or mir-382 on P56. (**c**) no change in mir-34a, mir-34c and mir-382 expression at P66-73. Data presented as means and standard errors of fold change. **p*<0.05. Relative to C.

**Fig S3.**

**
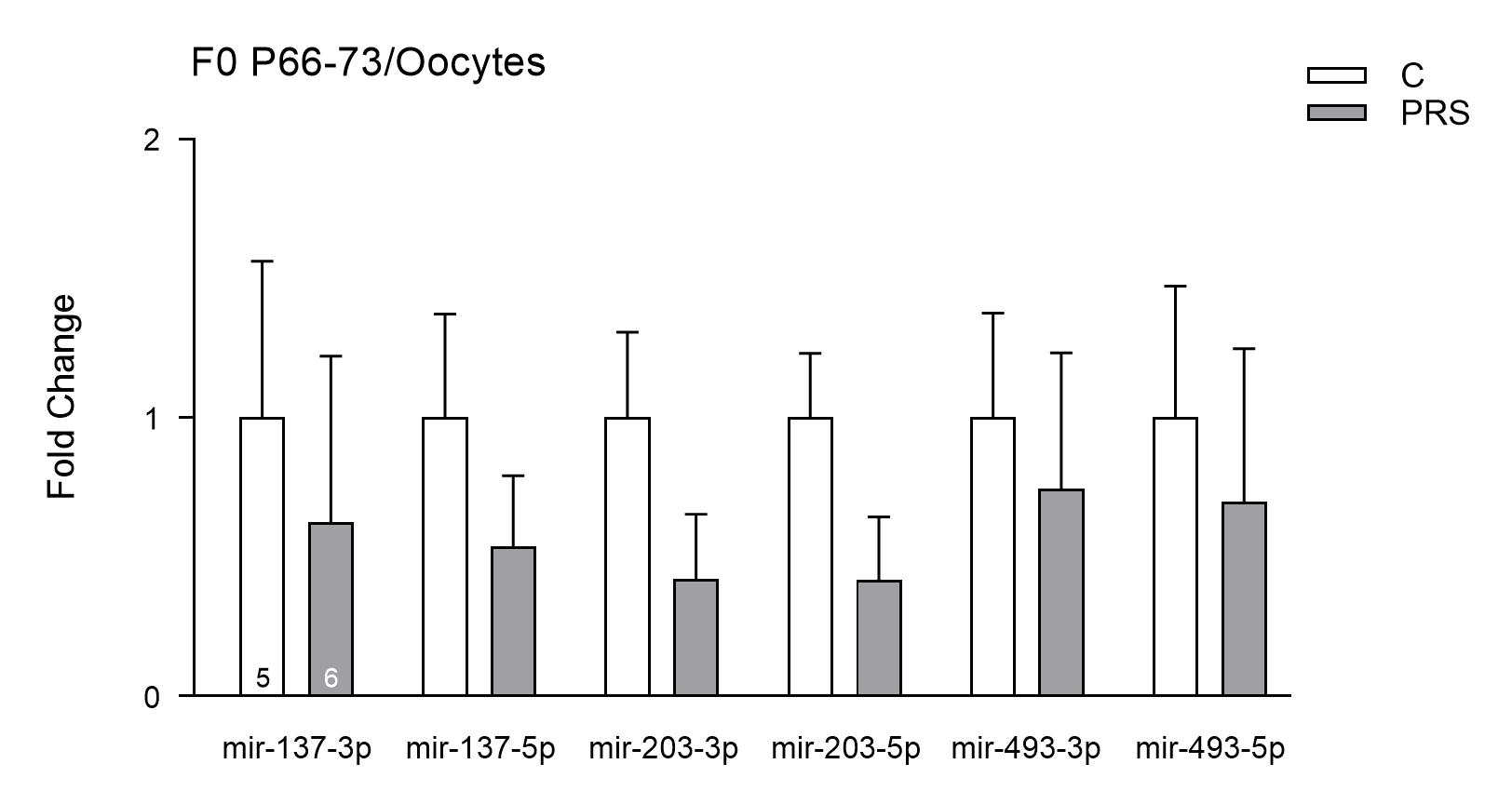
**

**Fig S3.** **miRNAs expression in oocytes of PRS-exposed and C females.** No PRS-induced changes were found in the expression of mir-137-3p, mir-137-5p, mir-203-3p, mir-203-5p, mir-493-3p and mir-493-5p.

**Fig S4.**

**
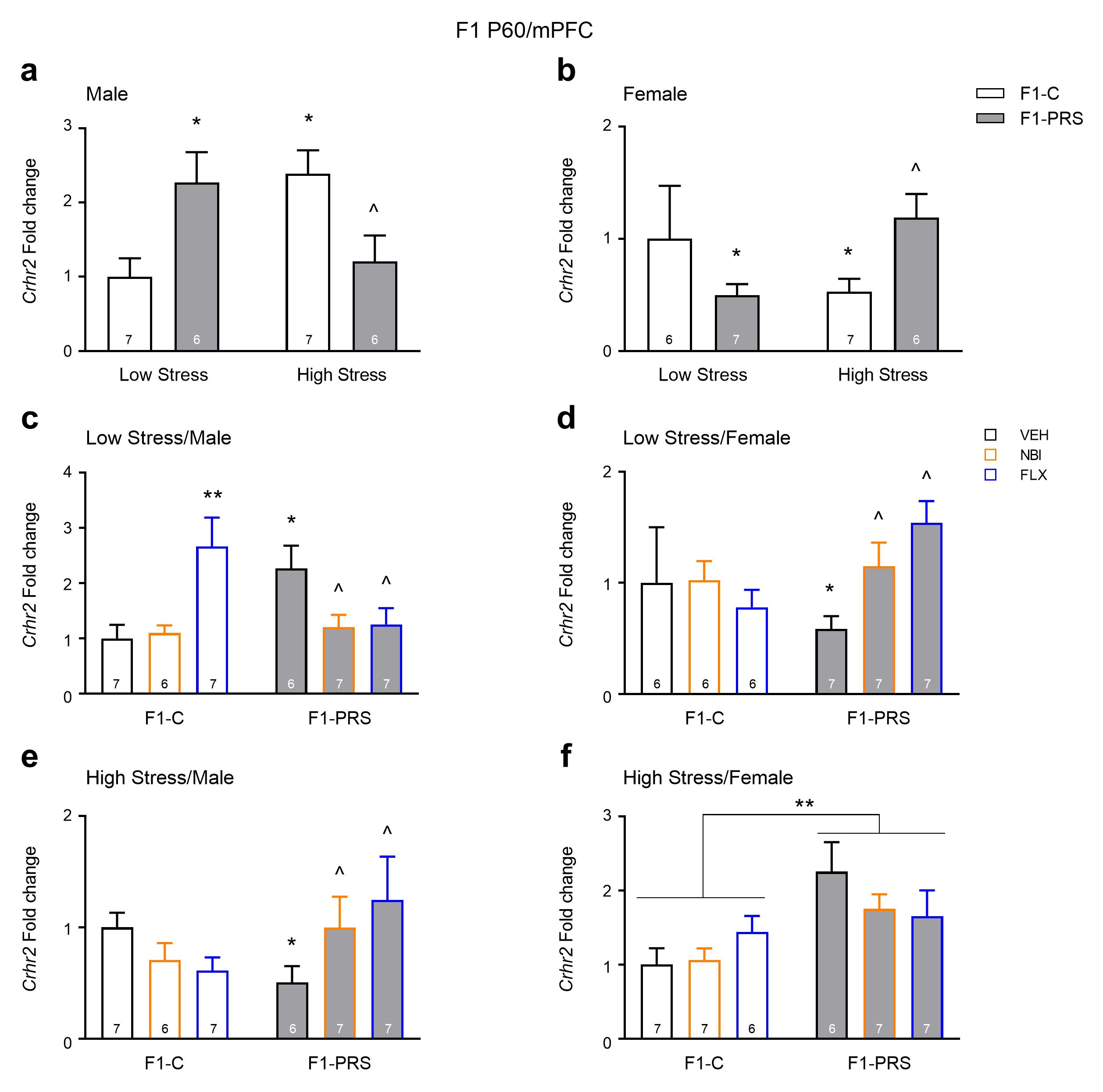
**

**Fig** **S4. Effects of maternal PRS, maternal drug treatment, and offspring exposure to stress on *Crhr2* mRNA expression in mPFC of adult F1 offspring.** (**a**) In F1-VEH males, high-stress test exposure increases *Crhr2* expression in F1-C rats, and maternal PRS increases *Crhr2* in low-stress but decreases it in high-stress rats (2x2 ANOVA, group x cohort F_1,22_=13.85, *p*<0.001). (**b**) In F1-VEH females, high-stress test exposure decreases *Crhr2* expression in F1-C rats, and maternal PRS decreases *Crhr2* in low-stress but increases it in high-stress rats PRS (group x cohort F_1,22_=13.55, *p*<0.001). (**c**) In low-stress males, maternal PRS increases *Crhr2* expression, and this is reversed by maternal NBI or FLX treatment. FLX increase *Crhr1* in F1-C rats (**c**, 2x3 ANOVA, group x drug F_2,34_=8.9, *p*<0.001). (**d**) In low-stress females, maternal PRS decreases *Crhr2* expression, and this is reversed by maternal NBI or FLX treatment (group x drug F_2,33_=6.59, *p*<0.01). (**e**) In high-stress males, maternal PRS decreases *Crhr2* expression, and this is reversed by maternal NBI or FLX treatment (group x drug F_2,34_=5.45, *p*<0.01). (**f**) In high-stress females, maternal PRS increases *Crhr2* expression, regardless of drug treatment (group F_1,34_=13.2, *p*<0.001). Data presented as means and standard errors. **p*<0.05. ***p*<0.001, post-hocs or main effects relative to C-VEH controls. ^*p*<0.05, post-hocs relative to high-stress F1-VEH or F1-PRS/VEH.

**Fig S5.**


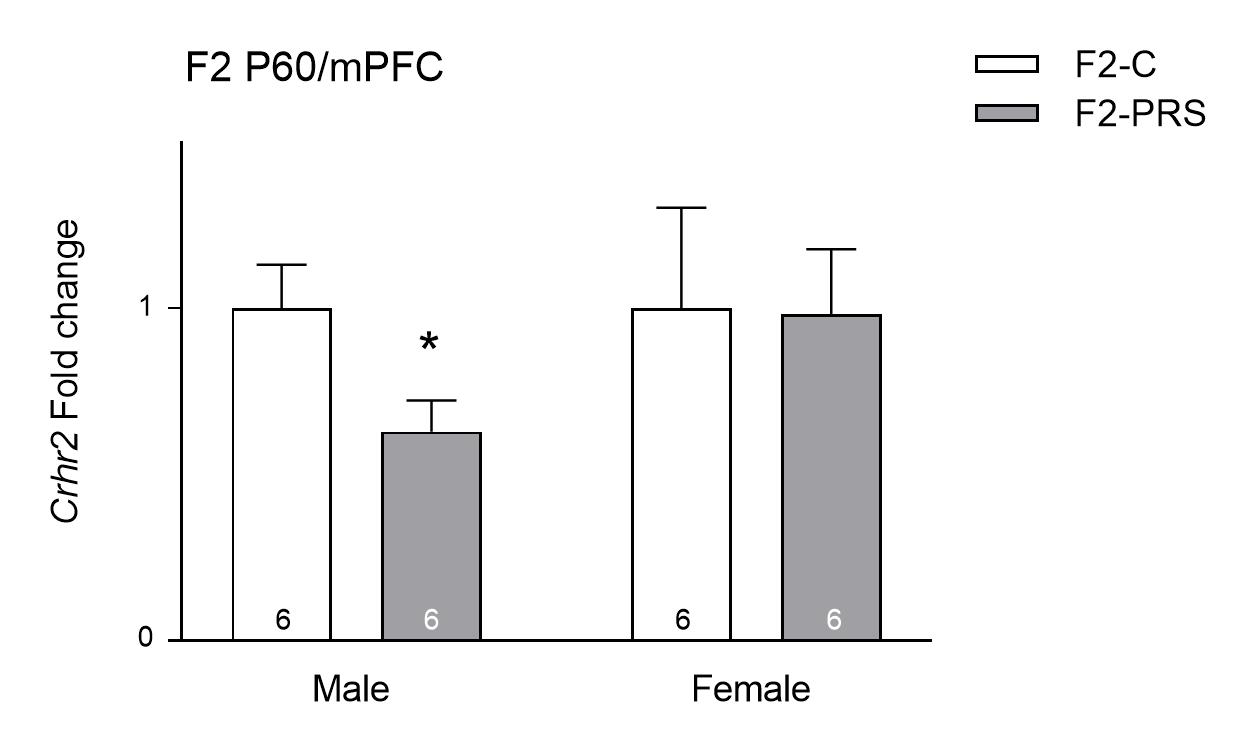


**Fig** **S5. PRS-induced changes in *Crhr2* expression in adult F2 offspring.** PRS decreases *Crhr2* mRNA expression in male, but not female, F2 offspring (one-way ANOVA, F_1,10_=5.4, *p*<0.05). Data presented as means and standard errors. **p*<0.05, relative to F2-C.

**Supplementary Statistical Analyses**

## **F1 adults: Behavior in low- and high-stress paradigms (supplementary analyses)**

**Behavior in low-stress paradigms: OF and NOR**

Male and female data were analyzed separately, since main effects of sex or interactions with the sex variable were observed in all tests (OF, time in center (s): sex x group interaction (2-Way ANOVA, F_1,147_=8.3, *p<*0.01). NOR, total exploration time (s) in the Test phase: sex x group interaction (3-Way ANOVA, F_1,138_=5.02, *p*<0.05). EPM, open arm latency (s): sex x group x drug interaction (3-Way ANOVA, F_2,140_=4.48, *p*<0.05). Fear Cond., mean freezing during tone presentation: sex (3-Way ANOVA, F_1,150_=4.1, *p*<0.05)).

In the NOR task, both male and female offspring explored the novel object more than the familiar one (repeated 2-Way ANOVA, object, males: F_1,67_=14.5, *p*<0.0001, females: F_1,63_=14.4, *p*<0.0001). Male offspring exhibited longer total exploration duration compared to females in the Sample Phase (F_1,140_=5.9, *p*<0.05), but maternal PRS or drug treatment did not affect the novelty ratio, the latency to approach either object or the frequency ratio (not shown).

**Behavior in high-stress paradigms: fear conditioning**

In the fear conditioning task, animals were excluded from the analysis if they exhibited stereotypic movements that interfered with FreezeFrame measurements, or due to technical problems (i.e., no shock delivered on acquisition day). Maternal PRS or drug treatment had no effect on stereotypic behavior (males, X^2^ (1,N=89)=8.9, *p=*0.12; females, X^2^ (1,N=91)=9.03, *p=*0.108), and mRNA or miRNA expression was not assessed in these animals. N’s and exclusion details are presented in **Table S1**.

On Day1 (Acquisition), pre-tone freezing was not affected by sex, maternal PRS or drug treatment. Freezing was assessed during the tone period (tone 1 onset to end of session) and during each tone. On Day 4, we again found freezing was extinguished over time in males (tone F_2.56,197.2_=66.7, *p*<0.001) and females (tone F_2.01,171.03_=25.5, *p*<0.001). No effects of maternal PRS or drug treatment were found in males. In females, F1-FLX and NBI-treated rats showed reduced overall freezing across PRS conditions (drug F_2,85_=7.0, *p*<0.01). On Day 5, there were low (<10%) freezing levels in all groups. Extinction across trials was observed in males (tone, F_2.8,220.1_=6.7, *p*<0.001). In females, offspring of FLX-and NBI-treated rats again showed reduced overall freezing compared to F1-VEH across all trials and PRS conditions (tone x drug F_6.5, 277.003_=2.3 *p*<0.05, post hoc *p’s*<0.05).

## **F2 adults: changes in behavior**

Male and female data were analyzed separately, since main effect of sex or interactions with the sex variable were observed in all tests (OF, center latency (s): sex x group interaction (2x2 ANOVA, F_1,49_=8.6, *p*>0.01). total locomotor activity: Females exhibited greater total locomotion than males (F_1,49_=7.5, *p*<0.01). Both males and females showed a decrease in locomotion over time (repeated-measures ANOVA of 5-min bins; males: time F_2,54_=176.7, *p<*0.0001; females: time F_2,44_=110.2, *p<*0.0001). We found no differences in frequency to enter the center in either males or females.

NOR, total exploration time (s) in the Test phase: sex x group (2x2 ANOVA, F_1,50_=7.05, *p>*0.05). SP, sociability index: sex (2x2 ANOVA, F_1,51_=4.78, *p>*0.05). EPM, duration: sex x group (2x2 ANOVA, F_1,52_=4.4, *p*>0.05); latency: sex x group (2x2 ANOVA, F_1,52_=7.2, *p*>0.01). Fear Cond., Pre-Tone Day 1: sex (2x2 ANOVA, F_1,37_=9.25, *p*>0.01).

**Fear conditioning**

Animals that exhibited stereotypic behavior (particularly stereotypic head movements) were excluded from the analysis. Grandmaternal PRS had no effect on stereotypic behavior in F2 offspring (males, X^2^ (1,N=30)=1.22,*p=*0.27; females, X^2^ (1,N=28)=0.48,*p=*0.83). N’s and exclusion details are presented in **Table S2.**

On Day 1, males froze more than females (2-Way ANOVA, F_2,30_=7.711, *p*<0.01). Freezing increased with each tone presentation in both males and females (tone, F_2,30_=32.9, *p*<0.0001, F_2,30_=42.67, *p*<0.0001, respectively). Pre-tone freezing and freezing during the first tone (prior to footshock) were not affected by grandmaternal PRS in either males or females (not shown). On Day 3, male and female offspring froze more during the tone period (tones 1-3) compared to period prior to tone onset (males, tone, F_1,24_=33.7, *p<*0.0001; female, tone, F_1,23_=24.2, *p*<0.0001).

**F1 and F2 adults: mRNA and miRNA expression changes**

Adult male and female data were analyzed separately, since interactions with the sex variable were observed in F1 for *Crhr1* (cohort x sex x group interaction (F_1,45_=12.09, p<0.001; **Fig 6**)) and *Crhr2* (cohort x sex x group interaction (F_1,44_=27.34, *p*<0.001; **Fig S4**)).

**References**

1. Council, N.R. *Guide for the care and use of laboratory animals* (National Academies Press, 2010).

2. Bock, J.*, et al.* Transgenerational sex-specific impact of preconception stress on the development of dendritic spines and dendritic length in the medial prefrontal cortex. *Brain structure & function* **221**, 855-863 (2016).

3. Leshem, M. & Schulkin, J. Transgenerational effects of infantile adversity and enrichment in male and female rats. *Developmental psychobiology* **54**, 169-186 (2012).

4. Shachar-Dadon, A., Schulkin, J. & Leshem, M. Adversity before conception will affect adult progeny in rats. *Dev Psychol* **45**, 9-16 (2009).

5. Zaidan, H. & Gaisler-Salomon, I. Prereproductive stress in adolescent female rats affects behavior and corticosterone levels in second-generation offspring. *Psychoneuroendocrinology* **58**, 120-129 (2015).

6. Zaidan, H., Leshem, M. & Gaisler-Salomon, I. Prereproductive stress to female rats alters corticotropin releasing factor type 1 expression in ova and behavior and brain corticotropin releasing factor type 1 expression in offspring. *Biological psychiatry* **74**, 680-687 (2013).

7. Shkolnik, K.*, et al.* Reactive oxygen species are indispensable in ovulation. *Proceedings of the National Academy of Sciences of the United States of America* **108**, 1462-1467 (2011).

8. Martinez, V., Wang, L., Rivier, J.E., Vale, W. & Tache, Y. Differential actions of peripheral corticotropin-releasing factor (CRF), urocortin II, and urocortin III on gastric emptying and colonic transit in mice: role of CRF receptor subtypes 1 and 2. *J Pharmacol Exp Ther* **301**, 611-617 (2002).

9. Yarushkina, N.I. & Filaretova, L.P. The peripheral corticotropin-releasing factor (CRF)-induced analgesic effect on somatic pain sensitivity in conscious rats: involving CRF, opioid and glucocorticoid receptors. *Inflammopharmacology* **26**, 305-318 (2018).

10. Hummel, M.*, et al.* Pain is a salient "stressor" that is mediated by corticotropin-releasing factor-1 receptors. *Neuropharmacology* **59**, 160-166 (2010).

11. Lee, H.J.*, et al.* Fluoxetine enhances cell proliferation and prevents apoptosis in dentate gyrus of maternally separated rats. *Mol Psychiatry* **6**, 610, 725-618 (2001).

12. Vorhees, C.V.*, et al.* A developmental neurotoxicity evaluation of the effects of prenatal exposure to fluoxetine in rats. *Fundamental and applied toxicology : official journal of the Society of Toxicology* **23**, 194-205 (1994).

13. Dryden, S., Brown, M., King, P. & Williams, G. Decreased plasma leptin levels in lean and obese Zucker rats after treatment with the serotonin reuptake inhibitor fluoxetine. *Hormone and metabolic research = Hormon- und Stoffwechselforschung = Hormones et metabolisme* **31**, 363-366 (1999).

14. Hodes, G.E., Yang, L., Van Kooy, J., Santollo, J. & Shors, T.J. Prozac during puberty: distinctive effects on neurogenesis as a function of age and sex. *Neuroscience* **163**, 609-617 (2009).

15. Lezak, K.R., Missig, G. & Carlezon, W.A., Jr. Behavioral methods to study anxiety in rodents. *Dialogues in clinical neuroscience* **19**, 181-191 (2017).

16. Choleris, E., Thomas, A.W., Kavaliers, M. & Prato, F.S. A detailed ethological analysis of the mouse open field test: effects of diazepam, chlordiazepoxide and an extremely low frequency pulsed magnetic field. *Neurosci Biobehav Rev* **25**, 235-260 (2001).

17. Winters, B.D., Saksida, L.M. & Bussey, T.J. Object recognition memory: neurobiological mechanisms of encoding, consolidation and retrieval. *Neuroscience & Biobehavioral Reviews* **32**, 1055-1070 (2008).

18. Ennaceur, A., & Delacour, J. A new one-trial test for neurobiological studies of memory in rats. 1: Behavioral data. *Behavioural Brain Research* **31**, 47-59 (1988).

19. Grayson, B., Idris, N.F. & Neill, J.C. Atypical antipsychotics attenuate a sub-chronic PCP-induced cognitive deficit in the novel object recognition task in the rat. *Behav Brain Res* **184**, 31-38 (2007).

20. Zaidan, H., Ramaswami, G., Barak, M., Li, J.B. & Gaisler-Salomon, I. Pre-reproductive stress and fluoxetine treatment in rats affect offspring A-to-I RNA editing, gene expression and social behavior. *Environmental Epigenetics* **4**, dvy021-dvy021 (2018).

21. Pellow, S., Chopin, P., File, S.E. & Briley, M. Validation of Open - Closed Arm Entries in an Elevated Plus-Maze as a Measure of Anxiety in the Rat. *Journal of Neuroscience Methods* **14**, 149-167 (1985).

22. Biedermann, S.V.*, et al.* An elevated plus-maze in mixed reality for studying human anxiety-related behavior. *BMC biology* **15**, 125 (2017).

23. Rao, R.M. & Sadananda, M. Influence of State and/or Trait Anxieties of Wistar Rats in an Anxiety Paradigm. *Annals of neurosciences* **23**, 44-50 (2016).

24. Jakovcevski, M., Schachner, M. & Morellini, F. Individual variability in the stress response of C57BL/6J male mice correlates with trait anxiety. *Genes, brain, and behavior* **7**, 235-243 (2008).

25. Maren, S. Neurobiology of Pavlovian fear conditioning. *Annu Rev Neurosci* **24**, 897-931 (2001).

26. Mahan, A.L. & Ressler, K.J. Fear conditioning, synaptic plasticity and the amygdala: implications for posttraumatic stress disorder. *Trends in neurosciences* **35**, 24-35 (2012).

27. Quirk, G.J. & Mueller, D. Neural mechanisms of extinction learning and retrieval. *Neuropsychopharmacol* **33**, 56-72 (2008).

28. Marek, R., Strobel, C., Bredy, T.W. & Sah, P. The amygdala and medial prefrontal cortex: partners in the fear circuit. *J Physiol* **591**, 2381-2391 (2013).

29. Vouimba, R.M. & Maroun, M. Learning-induced changes in mPFC-BLA connections after fear conditioning, extinction, and reinstatement of fear. *Neuropsychopharmacology* **36**, 2276-2285 (2011).

30. McEwen, B.S. Allostasis and allostatic load: implications for neuropsychopharmacology. *Neuropsychopharmacology* **22**, 108-124 (2000).

31. Ramachandra, R. & Subramanian, T. Neonatal Rat Brain. (London: CRC Press, 2011).

32. Paxinos, G., & Watson, C. *The Rat Brain in Stereotaxic Coordinates, Sixth Edition* (Academic Press, 2007).

33. Goren, S. & Dekel, N. Maintenance of meiotic arrest by a phosphorylated p34cdc2 is independent of cyclic adenosine 3',5'-monophosphate. *Biology of reproduction* **51**, 956-962 (1994).

34. Zaidan, H.*, et al.* A-to-I RNA editing in the rat brain is age-dependent, region-specific and sensitive to environmental stress across generations. *BMC genomics* **19**, 28 (2018).

35. Rozen, S. & Skaletsky, H. Primer3 on the WWW for general users and for biologist programmers. *Methods Mol Biol* **132**, 365-386 (2000).

36. Livak, K.J. & Schmittgen, T.D. Analysis of relative gene expression data using real-time quantitative PCR and the 2(-Delta Delta C(T)) Method. *Methods* **25**, 402-408 (2001).

37. Lewis, B.P., Burge, C.B. & Bartel, D.P. Conserved seed pairing, often flanked by adenosines, indicates that thousands of human genes are microRNA targets. *Cell* **120**, 15-20 (2005).

38. Betel, D., Wilson, M., Gabow, A., Marks, D.S. & Sander, C. The microRNA.org resource: targets and expression. *Nucleic acids research* **36**, D149-153 (2008).

39. Betel, D., Koppal, A., Agius, P., Sander, C. & Leslie, C. Comprehensive modeling of microRNA targets predicts functional non-conserved and non-canonical sites. *Genome biology* **11**, R90 (2010).

40. Grossman, H.*, et al.* Regulation of GVBD in mouse oocytes by miR-125a-3p and Fyn kinase through modulation of actin filaments. *Sci Rep* **7**, 2238 (2017).

41. Ninio-Many, L., Grossman, H., Shomron, N., Chuderland, D. & Shalgi, R. microRNA-125a-3p reduces cell proliferation and migration by targeting Fyn. *J Cell Sci* **126**, 2867-2876 (2013).

42. Pathan, M.*, et al.* FunRich: An open access standalone functional enrichment and interaction network analysis tool. *Proteomics* **15**, 2597-2601 (2015).
